# Composite receptive fields in the mouse auditory cortex

**DOI:** 10.1113/JP285003

**Published:** 2023-08-14

**Authors:** Sihao Lu, Grace W.Y. Ang, Mark Steadman, Andriy S. Kozlov

**Affiliations:** ^1^ Department of Bioengineering Imperial College London London UK

**Keywords:** auditory cortex, receptive field, sparse filtering, ultrasonic vocalizations, UMAP

## Abstract

**Abstract:**

A central question in sensory neuroscience is how neurons represent complex natural stimuli. This process involves multiple steps of feature extraction to obtain a condensed, categorical representation useful for classification and behaviour. It has previously been shown that central auditory neurons in the starling have composite receptive fields composed of multiple features. Whether this property is an idiosyncratic characteristic of songbirds, a group of highly specialized vocal learners or a generic property of sensory processing is unknown. To address this question, we have recorded responses from auditory cortical neurons in mice, and characterized their receptive fields using mouse ultrasonic vocalizations (USVs) as a natural and ethologically relevant stimulus and pitch‐shifted starling songs as a natural but ethologically irrelevant control stimulus. We have found that these neurons display composite receptive fields with multiple excitatory and inhibitory subunits. Moreover, this was the case with either the conspecific or the heterospecific vocalizations. We then trained the sparse filtering algorithm on both classes of natural stimuli to obtain statistically optimal features, and compared the natural and artificial features using UMAP, a dimensionality‐reduction algorithm previously used to analyse mouse USVs and birdsongs. We have found that the receptive‐field features obtained with both types of the natural stimuli clustered together, as did the sparse‐filtering features. However, the natural and artificial receptive‐field features clustered mostly separately. Based on these results, our general conclusion is that composite receptive fields are not a unique characteristic of specialized vocal learners but are likely a generic property of central auditory systems.

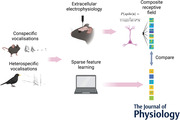

**Key points:**

- Auditory cortical neurons in the mouse have composite receptive fields with several excitatory and inhibitory features.
- Receptive‐field features capture temporal and spectral modulations of natural stimuli.
- Ethological relevance of the stimulus affects the estimation of receptive‐field dimensionality.

## Graphical abstract

**Abstract figure legend** We investigated how auditory cortical neurons in mice represent complex natural stimuli by presenting them with mouse ultrasonic vocalisations (USVs) and pitch‐shifted starling songs. Our findings revealed that neurons displayed composite receptive fields with several excitatory and inhibitory subunits, regardless of the natural vocalisation type. Using machine learning, we obtained statistically optimal features of these vocalisations and compared them to biological receptive‐field features using dimensionality reduction.

## 1 Introduction

Since it was first described in single optic nerve fibres of the frog (Hartline, 1938), the concept of the receptive field has proved an important tool to characterize neuronal functionality throughout the central nervous system. It refers to a region in sensory space that can modulate a neuron's activity. In the visual system, for example, this could correspond to a specific range of coordinates within the visual field within which brightness can modulate neuronal firing rate. In the auditory system, it might correspond to a particular range of frequencies and intensities. Early work describing receptive fields in mammals was performed in cats (Aitkin et al., 1975; Aitkin & Moore, 1975; Calford et al., 1983) where the selective tuning to a preferred frequency in neurons was shown, which was subsequently also shown in ferrets (Kowalski et al., 1996a, 1996b; Depireux et al., 2001), mice (Stiebler et al., 1997) and finally non‐human primates (deCharms et al., 1998).

One pervasive issue in characterizing neuronal receptive fields is the typically high dimensionality of the stimulus. To fully characterize the influence of each dimension on neuronal output, one would need to use impractically large stimulus sets and record responses over an unrealistically extended period. As a result, synthetic stimuli whereby only a small number of parameters of interest are manipulated have been typically used. A common example of this approach in the auditory system is the use of pure tones, played at a range of frequencies and intensities. Measuring the neuronal firing rate in response to each of these tones yields the pure‐tone receptive field, frequency response area or frequency tuning curve. Early experiments in the mouse using pure tones yielded important insights into the frequency‐to‐place mapping that occurs in the cochlea and is maintained throughout the auditory system (Stiebler et al., 1997). Later work explored the spectro‐temporal properties of neuronal receptive fields in different areas of the mouse auditory cortex using artificial, spectrally complex and temporally dynamic stimuli (Linden et al., 2003). Pure tones are still widely used to probe the auditory system, as more recently seen in the work by Li et al. (2015) investigating the cell‐type‐specific receptive‐field properties of neurons in the auditory cortex.

Experiments in rats using more complex sounds have indicated highly non‐linear response properties, meaning that it is difficult to predict a neuron's response to more complex stimuli by probing its response to individual pure tones (Machens et al., 2004). Therefore, it seems that the estimated receptive field is to a certain degree determined by the stimulus chosen to measure it (and, as discussed below, a method used to compute it). Given that one of the primary goals of sensory neuroscience is to understand the relationship between stimulus and behaviour, it is important to understand how neurons respond to behaviourally relevant, natural stimuli. In vocalizing animals (including humans), a strong candidate for such stimuli would be conspecific vocalizations. Prior studies on the processing of conspecific vocalizations have mainly focused on songbirds (Doupe, 1997; Theunissen et al., 2000; Grace et al., 2003; Kozlov & Gentner, 2016). There have been some studies done on ferrets (Harper et al., 2016a) and guinea pigs (Laudanski et al., 2012), but receptive fields have yet to be estimated using natural stimuli in mice.

Historically, there have been a number of barriers to successfully estimating receptive fields using complex, natural sounds. Firstly, traditional methods to characterize neuronal input–output relationships have been based on a number of assumptions that do not hold true in the case of natural stimuli. The spike‐triggered average (STA, or reverse‐correlation method (de Boer & Kuyper, 1968)), for example, requires that the stimuli are spherically symmetric (i.e. uniformly randomly sampled). In the auditory domain, such a stimulus is known as white noise, to which the auditory cortex appears to adapt leading to relatively weak responses over time (King et al., 2018). One approach to overcome the need to use white noise is to effectively ‘whiten’ the stimulus before carrying out reverse‐correlation analysis by compensating for intrinsic stimulus correlations (Theunissen et al., 2000). However, neurons in the neocortex can receive inputs from thousands of excitatory and inhibitory synapses of diverse origin simultaneously, so it seems likely that a receptive field characterized by a single spectro‐temporal ‘feature’ would often be insufficient to fully describe the neuronal input–output relationship. The spike‐triggered covariance (STC) is one method that yields a receptive field comprising multiple ‘features’ (each corresponding to a unique combination of stimulus dimensions that appears to modulate neuronal activity), but like reverse‐correlation, this method is also beset by a number of assumptions that do not hold true in the case of natural stimuli (Schwartz et al., 2006).

More recently, information‐theoretic approaches have been proposed to overcome the challenges described above and enable composite receptive‐field estimation from responses to natural sounds. The Maximally Informative Dimensions (MID) method (Sharpee et al., 2004) facilitates receptive‐field estimation by searching for a feature that maximizes the mutual information between the stimulus and the spikes. While this approach can be extended to estimate receptive fields with multiple features (like the STC (Rowekamp & Sharpee, 2011)), it is computationally intensive and limited by the impractical amount of physiological data required to estimate receptive fields comprising more than a small number of features.

An alternative approach is the maximum noise entropy (MNE) method (Fitzgerald et al., 2011). This method optimizes a model that maps the stimulus onto a logistic function, the output of which represents the probability of a spike. The parameters of this model are constrained by stimulus‐response correlations up to the second order (i.e. corresponding to overall firing rate, STA and STC), but it is otherwise as random and hence unbiased as possible. This method has been applied to neurons in the auditory forebrain of songbirds (Kozlov & Gentner, 2016), which showed that these neurons had composite receptive fields composed of multiple excitatory and inhibitory features.

The songbird auditory forebrain (specifically the caudo‐medial nidopallium, NCM) appears to play a particularly important role in processing birdsong and thus may have functionally diverged from the mammalian auditory cortex. More recently, Atencio and Sharpee (2017) applied the MNE model to characterize the responses of neurons in the cat auditory cortex, in response to dynamic moving ripples and ripple noise. That study also found that individual auditory neurons have multi‐dimensional receptive fields, albeit in response to artificial sounds. Another study, in the ferret auditory cortex, discovered multiple excitatory and inhibitory features in single neurons driven by an ensemble of natural sounds comprising environmental sounds, ferret vocalizations and human speech (Harper et al., 2016b). An emerging theme from these studies is that central auditory neurons have composite receptive fields, and that their properties may differ depending on whether they are probed with artificial or natural sounds.

In this study, we wanted to answer the following questions: When auditory cortical neurons in the mouse, a powerful model organism in neuroscience, respond to ethologically relevant mouse vocalizations (USVs), what is the dimensionality of the feature space? Does it differ depending on whether neurons are stimulated with ethologically relevant sounds (mouse USVs) or with natural, but ethologically irrelevant complex sounds (pitch‐shifted birdsongs)? Finally, how do these features compare to those learned by an artificial neural network optimized for sparse representation?

## 2 Materials and methods

### 2.1 Ethical statement

All procedures were carried out under the terms and conditions of licences issued by the UK Home Office under the Animals (Scientific Procedures) Act 1986. Adult female C57BL/6 mice (Charles River, Margate, UK) were used for electrophysiological recordings (*N* = 25, aged 5–27 weeks). All mice were housed in groups of five and allowed food and water *ad libitum*. Animals were humanely killed in accordance with Schedule 1 to the Animals (Scientific Procedures) Act 1986.

### 2.2 Surgical preparation

Animals were anaesthetized using a mixture of fentanyl, midazolam and medetomidine (0.05, 5 and 0.5 mg/kg, respectively). A midline incision was made over the dorsal surface of the cranium, and the left temporalis muscle was partially resected. The location on the skull over the left auditory cortex was identified using a rostro‐caudal coordinate of 70% bregma‐lambda and a dorso‐ventral coordinate of bregma −2.2 mm (the lateral coordinate being determined by the surface of the skull). A steel headplate comprising a bent piece of flat bar (approximately 5 mm × 30 mm) was attached to the dorsal surface of the skull using a combination of tissue adhesive (Histoacryl) and dental cement. This was subsequently used in combination with a magnetic stand to secure the animal in place for the remainder of the surgery and recordings.

A small (⌀ = 2mm) craniotomy was made over the auditory cortex using a dental drill and burr. For recordings made using multishank tetrode arrays, an incision was made in the dura using a small needle, through which the probes were later inserted. For single shank polytrode recordings, the dura was left intact before insertion. The surface of the brain was protected from desiccation by regular application of warmed saline solution or agar. A second craniotomy was made approximately over the contralateral motor cortex, such that a machine screw (M1 × 2 mm) could be secured into the bone to provide a reference signal. The animal was then transferred to an acoustic chamber, where the head was again secured by means of the headpost and a magnetic stand and body temperature was maintained throughout the duration of the experiment using activated Deltaphase isothermal pads. Throughout the entirety of the surgical procedure and the electrical recording, the depth of anaesthesia was continuously monitored through the breathing rate and periodic assessment of the toe pinch reflex.

### 2.3 Electrophysiological recording

Extracellular signals were recorded using silicon multielectrode probes (Neuronexus). Two styles of probe were used to obtain the data presented here: a 4‐shank, 4 by 2 tetrode array and a single‐shank, 32‐channel polytrode. The tetrode array facilitated simultaneous recordings at sites spanning the tonotopic map, whereas the polytrode provided greater robustness to electrode drift. For both tetrodes and polytrodes, the conductive site areas were 177μm^2^. Signals were amplified and digitized at a sample rate of 40 kHz using a Plexon Omniplex Neural Recording Data Acquisition System (Plexon Inc.). A subset of the recordings was acquired through a Neuronexus Smartbox Pro (Neuronexus) at 30 kHz.

### 2.4 Stimuli

Mouse ultrasonic vocalizations (USVs) were obtained from mouseTube (Torquet et al., 2016), an annotated database of ultrasonic mouse calls. The database was searched for vocalizations produced by male C57BL/6 mice. These were downloaded and individual song bouts were extracted manually using Audacity. The extracted bouts were ramped on and off using a 10 ms cosine squared ramp and concatenated into a single audio file with a sample rate of 250 kHz. Two versions of the USV stimulus were produced. The first had a total duration of 3 min and 30 s. The second version was edited such that periods of silence or movement noise between song bouts were shortened. This stimulus had a total duration of 2 min.

**Figure 1:**
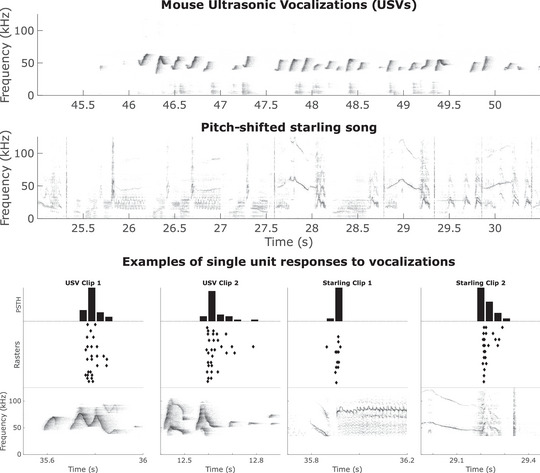
Examples of stimuli and responses The top panel shows a spectrogram of a 5.5 s fragment of a mouse ultrasonic vocalization stimulus. The second panel shows a spectrogram of a 5.5 s fragment of a starling song, pitch‐shifted up by a factor of 10 to better match the overall spectrum of the mouse USVs. The bottom panel shows short, 0.5 s fragments of both the USV and starling stimuli alongside the corresponding spiking response. These plots comprise the spectrogram of the stimulus, a raster plot of spiking responses whereby each dot represents a single spike, the *x*‐coordinate corresponding to the time and the *y*‐coordinate corresponding to the stimulus repetition number. Above the raster is a post‐stimulus time histogram (PSTH).

In addition, a stimulus based on starling vocalizations was produced. Starling song recordings were taken from the database produced by Kozlov & Gentner (2016). Briefly, spontaneously produced songs of adult male European starlings (*Sturnus vulgaris*) were recorded in a sound attenuation chamber at 44.1 kHz. These were then upsampled to 250 kHz and pitch‐shifted up by a factor of 10 to better match the power spectra of the mouse vocalizations. Similar to the mouse vocalizations, two versions of the starling stimuli were used: the first had a total duration of 3 min 30 s and the second was more condensed, with periods of silence removed and had a total duration of 2 min. All audio files were adjusted such that the RMS intensities were matched. An example of both these stimuli can be seen in the top two panels of Fig. 1.

For experiments using pure tone stimuli, we played a randomized sequence of 26 tones, linearly spaced between 30 and 90 kHz. Each tone was 200 ms in duration and occurred 5 times in the sequence.

Stimuli were presented for a minimum of 10 repetitions, up to a maximum of 20 repetitions, using an Avisoft UltraSoundGate Player 116 (Avisoft Bioacoustics, Glienicke, Germany) connected to a PC. In all cases, the sound level was adjusted such that the peak intensity did not exceed 85‐dB SPL. Playback was monitored using an UltraSoundGate 166H coupled with an Avisoft‐Bioacoustics CM16/CMPA condenser microphone connected to the same PC. Signal intensity was monitored in dB SPL by calibrating the system using a calibrated 40‐kHz reference signal generator (Avisoft Bioacoustics). To synchronize neural responses and the presented stimuli, a digital output from the Player 116 was connected to a digital input in the data acquisition system, which triggered an event at the onset of stimulus playback.

### 2.5 Spike sorting

Spikes were sorted offline either using a semi‐automated principle components clustering approach implemented in Plexon Offline Sorter (Version 4.4.0) or Kilosort3 (Steinmetz et al., 2021). For the Plexon Offline Sorter, spike events were detected by setting a threshold close to the noise floor on each electrode. Detected events were then sorted and classified as either single‐ or multiunit. The criteria for classifying a unit as single were that there was a locally consistent spike shape where the distribution of peak amplitudes was distinct from the spike detection threshold. Clustering was performed using the built‐in k‐means algorithm, the results of which were inspected and corrected visually using 3‐D visualizations of a number of parameters including the first three principle components, maxima, minima and overall energy of the detected spike events. As extracellular responses at a single recording location were recorded over a time period of up to 2 h, some recordings exhibited electrode drift, whereby spike shapes changed gradually over time. Therefore, all sorting was carried out using 3‐D clusters versus time visualization. Spikes extracted using the automatic spike sorting algorithm Kilosort3 were manually curated with Phy (Rossant & Harris, 2013). Units were considered well‐isolated single units if they showed clear refractory periods in their autocorrelogram and had fewer than 1% spikes within a 1 ms inter‐spike interval.

### 2.6 Receptive‐field characterization

The receptive fields of single units were characterized using the MNE method (Fitzgerald et al., 2011). Briefly, this method comprises fitting a model that describes the probability of a spike as a function of a stimulus, **s**, as $P\left(spike|s\right)=(1+exp{\left(a+sh+s^{T}Js\right)}^{-1}$, where *a*, *h* and *J* are parameters optimized via gradient descent. These parameters are determined such that the predicted firing rate, STA and STC match those in the observed data. In addition, we computed the receptive‐field features of single units using the STC method. This method considers a segment of the stimulus directly preceding a spike. The length of the segment is chosen to match the parameters used for the MNE method. The receptive‐field features of the unit are then determined to be the eigenvectors of the covariance matrix of all such segments (Schwartz et al., 2006).

The choice of how to represent the stimulus can have a profound effect on the success of the model. For auditory data, the most straightforward representation would be the sample values of the acoustic waveform. However, this would yield a stimulus representation with intractably large dimensionality. It is, therefore, common to represent an auditory stimulus in terms of a spectrogram. The frequency limits of the spectrogram were determined through a combination of bioacoustical analysis of the stimulus set and published information on the behavioural audiogram of C57BL/6 mice.

Bioacoustical analysis of the USV stimuli was carried out using Avisoft SAS Lab Pro (Version 5.2.09). Spectrograms of the entire USVs corpus were generated using a 512‐point Hann window and 50% overlap. Individual USV syllables were detected and analysed using a semi‐automatic thresholding process, whereby a threshold of −50 to −45 dB relative to the maximum intensity (with a hold time of 10 ms) was used. This method would occasionally erroneously detect broadband, movement‐related sounds, or else combine elements occurring in rapid succession, so detected elements were manually inspected and corrected where necessary. Whistle contours were then extracted by measuring the frequency of the highest intensity at intervals of 5 ms throughout each element. This analysis yielded a database of 1616 individual USV syllables with a mean duration of 70.6 ms (σ = 55 ms) and overall average frequency of 55.4 kHz. To determine a suitable frequency range that covers the majority of the vocalization spectra, the minimum and maximum frequencies (fmin and fmax, respectively) of each whistle contour were extracted. A lower frequency bound was set to two standard deviations below the mean fmin (27.3 kHz). An upper bound was set to three standard deviations above the mean fmax (100.6 kHz). Similarly, a maximum lag was set to two standard deviations above the mean duration (*μ = 70.6* ms, σ=55 ms), and rounded to the nearest bin size (180 ms). As the behavioural audiogram of the mouse extends to lower frequencies, a small number of broader, low frequency bins were also included. Spectrograms were generated from the audio files using the Matlab function spectrogram. This was calculated using a 1024‐point Hann window and a 50% overlap, yielding a representation with frequency bins separated by 244.1 Hz. A set of edge frequencies was defined, within which frequency bins were averaged. These were logarithmically spaced such that the resulting representation had six low frequency bins spanning from 18.1 to 27.3 kHz and 26 high frequency bins spanning 27.3–100.6 kHz. A temporal bin size of 15 ms was used. Columns of the spectrogram corresponding to times falling within each 15‐ms bin were also averaged together. The intensity of the resulting spectrogram was then converted to a dB scale.

Because for each temporal bin, the spectro‐temporal receptive field (STRF) would comprise the spectrogram values at the same time and also during the preceding 180 ms, the spectrogram was concatenated with temporally lagged version of itself, so each row now comprised the dB intensity values in each of the 32 spectral bins, followed by the intensity values on the spectral bins at the previous timesteps, up to the maximum lag. This resulted in an M×N stimulus matrix, where *M* is the total number of time steps and *N* is equal to the number of spectral bins multiplied by the number of timesteps in the STRF, in this case equal to 384 (32 spectral bin multiplied by 12 timesteps). For the response, a post‐stimulus time histogram (PSTH) was calculated using the same temporal bins and smoothed using a 225 ms Hanning window.

The MNE model for each unit was trained with 80% of the stimulus‐response data. The remaining 20% of the data were used to test the model. The training data were randomly extracted from the full recording without replacement to minimize the effects of any non‐stationarity of the data. After training the MNE model for each unit, the model was used to predict a response to the test stimulus. The correlation coefficient between the model predictions and the recorded neural activity was calculated to assess the performance of the model.

Neural activity of a single unit is not perfectly correlated between repeated presentations of the stimulus; this affects how the performance of a model based on its correlation coefficient with recorded data is interpreted. To account for this inherent variability in the neural activity, we estimate the expected correlation coefficient between the responses to repeated presentations of the same stimulus in the test set (Hsu et al., 2004; Touryan et al., 2005). The expected correlation coefficient ($r_{A,{\bar{R}}_{M}}$) is defined as the correlation between the true firing rate, *A*, and the firing rate (${\bar{R}}_{M}$) as measured by averaging over *M* repeats. As detailed in Hsu et al. (2004), we can calculate $r_{A,{\bar{R}}_{M}}$ using the following equation:

$$r_{A,{\bar{R}}_{M}}^{2}=\frac{2}{1+\sqrt{\frac{1}{r_{{\bar{R}}_{M1},{\bar{R}}_{M2}}^{2}}}},$$

where $r_{{\bar{R}}_{M1},{\bar{R}}_{M2}}$ is the correlation between ${\bar{R}}_{M1}$ and ${\bar{R}}_{M2}$. ${\bar{R}}_{M1}$ is the firing rate estimated by averaging over M/2 repetitions and ${\bar{R}}_{M2}$ is the firing rate estimated by averaging over the other M/2 repetitions of the neural response.

The correlation coefficients between the model prediction and the test set were then scaled by the expected correlation of the test set. Both raw and scaled correlation coefficients were plotted for each unit.

### 2.7 Feature estimation

To construct a compact representation of both the starling songs and mouse USVs, we used sparse filtering (Ngiam et al., 2011) to learn a set of basis features subject to the following constraints: populations sparsity, lifetime sparsity and high dispersal. Population sparsity specifies that only a small subset of features should be active at a given time, while lifetime sparsity specifies that each individual feature should be minimally active. High dispersal means that all the features have a similar amount of activity. One can compare this property with divisive normalization in neural circuits. These three constraints are typically observed in the neural activity in the cortex. How these constraints are applied is specified in Ngiam et al. (2011). Briefly, sparse filtering aims to find features based on their activity defined as F=W∗X where *F* is the activity matrix which gives the activity of the features in *W* for each of the examples given in *X*. Each row ***w***_*i*_ of *W* is one feature, and each column ***x***_*j*_ of *X* is one example. Thus each element $f_{ij}$ in *F* defines the activity of feature ***w***_*i*_ for example ***x***_*j*_. The algorithm minimizes a cost function which takes the activity matrix as input by iteratively changing the features in *W* until an optimal set of features is found. Sparse filtering requires a single hyperparameter, the number of features to compute. For our analyses, we set the number of features to 128.

### 2.8 Latent space projection

To compare (1) the receptive‐field features obtained from the neural activity with (2) the features estimated from the stimulus using the sparse filtering method as well as with (3) syllables segmented from the raw stimulus, we project the features into a latent space using the UMAP algorithm (McInnes et al., 2018). The UMAP algorithm finds low‐dimensional representations and has been shown to distinguish successfully between vocalizations of various animal species (Sainburg et al., 2020).

The syllables used in the projection were manually segmented from the stimulus, which resulted in 716 USV syllables and 1010 starling syllables. From this set of syllables, we excluded any USV syllables shorter than 90 ms and any starling syllables shorter than 150 ms. This resulted in a final set of 552 USV syllables and 388 starling syllables. Spectrograms of the individual syllables were created using the same parameters as described in Section 2.6. Both the MNE features and the features estimated with sparse filtering were zero‐padded to match the size of the spectrogram of the longest individual syllable. In addition to the segmented syllables and features obtained from MNE and sparse filtering, we added randomly generated noise in the form of spectrograms with the same number of frequency bins as the other inputs and a random time bin, up to the longest segmented syllable. The distribution of values in the noise spectrograms was chosen to be uniform. Before approximating the latent space, the modulation power spectra of all the inputs were calculated by taking the two‐dimensional Fourier transform of the spectrograms. The resultant projections in the latent space were clustered using the HDBSCAN algorithm (McInnes & Healy, 2017) which has been shown to extract clusters reliably in UMAP embeddings.

## 3 Results

### 3.1 Mouse auditory cortical neurons have composite receptive fields

Neuronal spiking activity in response to acoustic stimulation with mouse USVs was recorded from a total of 74 single units in the left auditory cortices of 25 anaesthetized mice. We also recorded responses to pitch‐shifted starling song in a subset of these units (*n* = 54). Figure 1 shows examples of the recorded spikes during stimulation of both mouse USVs and pitch‐shifted starling songs.

**Figure 2:**
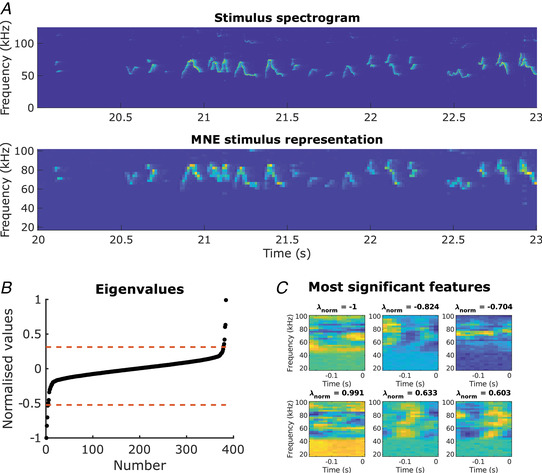
Estimated receptive‐field features *A*, The top panel shows a spectro‐temporal representation of a section of the stimulus. The second panel shows the same segment after reducing the temporal resolution and logarithmically spacing the frequency bins; this representation was used to train the maximum noise entropy model. *B*, The eigenvalues of all the features along with the significance thresholds (dotted line) for an example unit. The features associated with the eigenvalues beyond the significance bounds are considered to be significant. *C*, The three most significant excitatory (top row) and inhibitory (bottom row) features with their associated eigenvalues ($\lambda_{norm}$). The eigenvalue determines the feature's contribution to the composite receptive field. [Colour figure can be viewed at wileyonlinelibrary.com]

**Figure 3:**
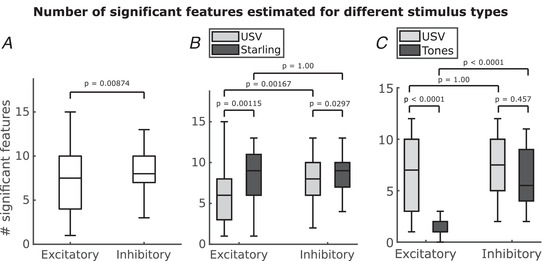
Number of significant features estimated for different stimulus types *A*, Number of significant excitatory (μ=6.85, σ=3.71) and inhibitory features (μ=8.17, σ=2.29) for each of the receptive fields estimated. P=0.00874 (Wilcoxon rank sum test). *B*, Comparison between the number of significant features when the MNE model is trained with responses to USVs (n=74) *versus* responses to pitch‐shifted starling songs (n=54). (Wilcoxon rank sum test, Bonferroni–Holm multiple comparison correction.) Every unit stimulated with the starling songs was also stimulated with USVs. *C*, Number of excitatory (μ=6.44,σ=3.3) and inhibitory (μ=7.29,σ=2.68) features when the MNE model is trained with responses to USVs *versus* number of excitatory (μ=1.38,σ=0.99) and inhibitory (μ=6.18,σ=2.99) features of models trained on responses to pure tones (n=34). (Wilcoxon rank sum test, Bonferroni–Holm multiple comparison correction.) Data shown in (*C*) are from different units than those shown in (*B*). Each unit in (*C*) was presented with both USVs and pure tones.

The receptive fields produced by the MNE method model yield a single linear feature (analogous to the STA) and a number of quadratic features (analogous to the STC). Figure 2 shows examples of the three most significant quadratic excitatory and inhibitory features of a single unit alongside their normalized eigenvalues indicating their relative contribution to the composite receptive field. A previously reported statistical method (Kozlov & Gentner, 2016) was employed to determine a number of significant excitatory and inhibitory features for each unit (Fig. 2 *B*). The numbers of excitatory and inhibitory features for all the units are summarized in Fig. 3 *A*. On average, the composite receptive fields comprised more significant inhibitory features (μ = 8.17, σ = 2.29) than excitatory ones (μ = 6.85, σ = 3.71). A Wilcoxon rank sum test confirmed that this difference was statistically significant (P=0.00874). Indeed, this increased number of inhibitory features is apparent even when fitting the models to responses to pure tone stimuli, even though significantly fewer excitatory features were found when using pure tone stimuli (Fig. 3 *C*). This result points towards the possibility that the dimensionality of responses depends on whether simple or complex stimuli were used to probe the receptive field of the cell.

### 3.2 Receptive fields estimated from con‐ and heterospecific vocalizations

In addition to mouse USVs, responses to pitch‐shifted starling song of matched duration were recorded in 54 of the single units to understand better the importance of ethological relevance of the stimulus to estimating receptive fields. The receptive fields of these units were estimated using the two stimuli separately.

A comparison between the number of significant features estimated from conspecific and heterospecific vocalizations is shown in Fig. 3 *B*. We show that for the conspecific stimulus, the difference between the number of significant excitatory features (μ=5.86, σ=3.59) and the number of significant inhibitory features (μ=7.70, σ=2.31) is significant (P=0.00167, Wilcoxon rank sum test, Bonferroni–Holm multiple comparison correction). On the contrary, there is no significant difference between the number of excitatory features (μ=8.20, σ=3.47) and the number of inhibitory features (μ=8.81, σ=2.11) estimated from the heterospecific stimulus (P=1.00, Bonferroni–Holm multiple comparison correction). In addition, we show that the number of significant excitatory features differs between models trained using the conspecific stimulus and those trained using the heterospecific stimulus (P=0.00115, Wilcoxon rank sum test, Bonferroni–Holm multiple comparison correction). Likewise, there is a difference between the number of significant inhibitory features (P=0.00297, Wilcoxon rank sum test, Bonferroni–Holm multiple comparison correction).

**Figure 4:**
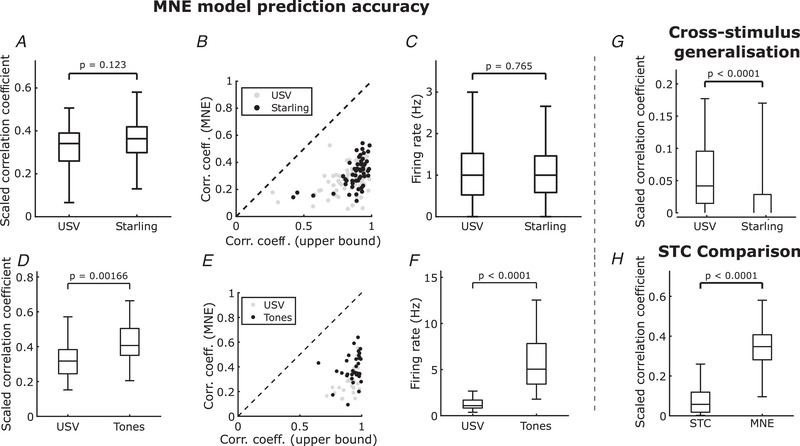
MNE model prediction accuracy and generalization performance *A*, Correlation coefficients between MNE model predictions and recorded neural activity in response to stimulation with either USVs (n=74, μ=0.33, σ=0.11) or pitch‐shifted starling songs (n=54, μ=0.36, σ=0.10). P=0.123 (Wilcoxon rank sum test). The correlation coefficients are scaled by the correlation coefficient between the responses to repeated presentations of the same stimulus in the test set. *B*, Model prediction correlation coefficients plotted as a function of the expected correlation between trials to indicate an upper bound on the correlation coefficient achievable by the model. Two sets of points are shown; USVs (grey) and pitch‐shifted starling song (black). Dashed line indicates a gradient of 1. *C*, Comparison of the firing rates of the recorded units in response to either USVs (n=74, μ=1.3 Hz, σ=1.2 Hz) or pitch‐shifted starling songs (n=54, μ=1.3 Hz, σ=1.2 Hz). P=0.765 (Wilcoxon rank sum test). In both cases, the firing rates were calculated using full recordings and not only test data. *D*, Correlation coefficients between MNE model predictions and recorded neural activity in response to stimulation with either USVs (n=34, μ=0.32, σ=0.10) or pure tones (n=34, μ=0.42, σ=0.13). P=0.00166 (Wilcoxon rank sum test). The correlation coefficients are scaled by the correlation coefficient between the responses to repeated presentations of the same stimulus in the test set. *E*, Same as (*B*), but data shown are for predictions based on models trained on USVs (grey) or pure tones (black). *F*, Comparison of the firing rates of the recorded units in response to either USVs (n=34, μ=1.4 Hz, σ=1.2 Hz) or pure tones (n=34, μ=6.5 Hz, σ=5.2 Hz). P<0.0001 (Wilcoxon rank sum test). In both cases, the firing rates were calculated using full recordings and not only test data. USV data used for (*D*), (*E*) and (*F*) are not included in (*A*), (*B*) and (*C*). *G*, Prediction accuracy across stimulus types given by correlation coefficient of predicted neural activity to other stimulus type. *USV* label denotes models trained with responses to USVs predicting responses to starling songs (n=54, μ=0.052, σ=0.070), and *Starling* label denotes models trained on starling song responses predicting responses to USVs (n=54, μ=0.0081, σ=0.094). P<0.0001 (paired *t* test). *H*, Comparison between the prediction accuracy of STC (n=128, μ=0.077, σ=0.077) and MNE (n=128, μ=0.34, σ=0.11) models. The scaled correlation coefficients for models fitted to both stimulus types were considered. P<0.0001 (paired *t* test).

**Figure 5:**
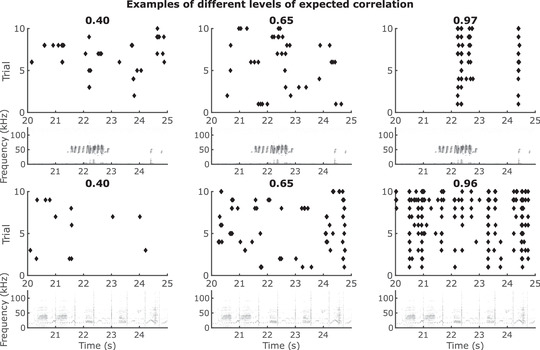
Examples of different levels of expected correlation Raster plots showing three different levels of expected correlation. (Top) shows the expected correlation for three units responding to USVs. The expected correlation for each unit is indicated above the raster plot. The spectrogram of the associated stimulus is plotted below each of the raster plots. (Bottom) shows the expected correlation for three units responding to starling song. The expected correlation for each unit is indicated above the raster plot. The spectrogram of the associated stimulus is plotted below each of the raster plots. Note that the units in (top) and (bottom) are not the same.

Figure 4 *A* shows that the spike rate prediction, as measured by the scaled correlation coefficient, did not differ significantly between units stimulated with mouse USVs and units stimulated with pitch‐shifted starling songs (P=0.123, Wilcoxon rank sum test). The relation between the measured correlation coefficient between the model prediction and the recording and the expected correlation coefficient of the associated unit is shown in Fig. 4 *B*. Raster plots for different levels of expected correlation can be seen in Fig. 5. To account for any differences in firing rate between the responses from mouse USVs and pitch‐shifted starling songs, the firing rates of all recorded responses in both stimulus paradigms were compared, as shown in Fig. 4 *C*. No significant difference was observed between the responses to conspecific and heterospecific vocalizations (P=0.765, Wilcoxon rank sum test). In addition, we show that the MNE method significantly outperforms the widely used STC method (Fig. 4 *H*, P<0.0001, paired *t* test). Lastly, we studied the generalization ability of MNE models trained on USVs and starling songs using models trained on one to predict responses to the other stimulus type. The predictive power decreased significantly when the model predicted responses to the other stimulus type, especially for models trained on pitch‐shifted starling songs as their cross‐stimulus response prediction was worse than that of the models trained on USVs (Fig. 4 *G*, P<0.0001, paired *t* test).

### 3.3 Stimuli modelled with basis features

**Figure 6:**
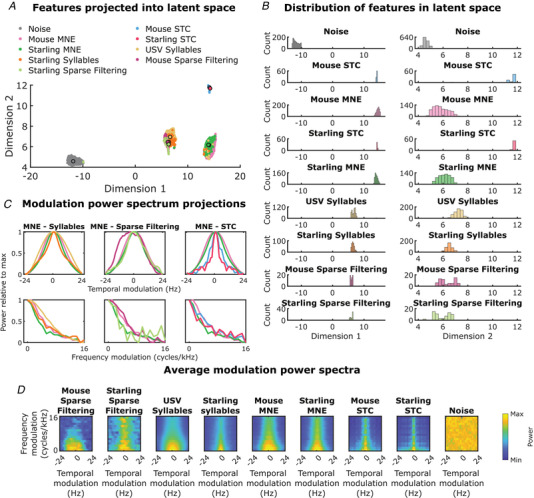
Analysis and comparative visualization of features *A*, UMAP projections onto two dimensions of: segmented syllables present in USVs and pitch‐shifted starling songs, significant features estimated using both the STC method and the MNE models, features estimated using the sparse filtering method and randomly generated noise. Larger points with outlines are the projection of the mean modulation power spectrum of the class as indicated by the label colour. *B*, Histograms of each of the different categories in dimension 1 (left) and dimension 2 (right). *C*, Projections of modulation power spectra along the temporal axis (top) and spectral axis (bottom). Projections are all plotted as power relative to the maximum to facilitate comparison. The columns show the modulation spectra of MNE features in comparison with the syllables, sparse filtering features and STC features, respectively. Colours are the same as in (*A*). *D*, Mean modulation power spectra for each of the classes in (*A*). [Colour figure can be viewed at wileyonlinelibrary.com]

**Figure 7:**
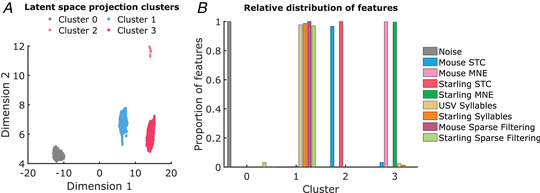
Clustering and distribution analysis of features *A*, Clustering UMAP projections of: segmented syllables present in USVs and pitch‐shifted starling songs, significant features estimated using both the STC method and the MNE models, features estimated using the sparse filtering method, and randomly generated noise. Four clusters were detected and are labelled. Cluster 0 clearly separates the noise from the other features, while cluster 1 includes the USVs and starling‐song syllables segmented from the stimuli and the sparse filtering features estimated from the stimuli. Lastly, both the STC and the MNE features are separated into their own respective clusters. *B*, Relative distribution of each class to the four detected clusters. [Colour figure can be viewed at wileyonlinelibrary.com]

We calculated a set of optimal basis features for both the mouse USVs and starling songs using the sparse filtering algorithm to determine whether receptive fields are formed to represent the stimulus in a statistically optimal way. For both sets of stimuli, 128 features were calculated. Sparsity of the population activity of the features was confirmed by calculating the percentage of features with activity lower than $\frac{1}{e}*f_{max}$, where $f_{max}$ is the maximum activation across the entire stimulus. This was 95.2 and 92.6% for the features estimated from USVs and the starling song, respectively. We then compared these calculated features with the estimated biological receptive fields and with the syllables from the raw stimulus from which they were calculated to determine the similarity between them. To do this, we projected them into a latent space using the UMAP algorithm (see Methods). The resulting projection is shown in Fig. 6 *A*,*B*. While the MNE features obtained with both the conspecific (USVs) and heterospecific (starling songs) stimuli formed a single cluster, only a small subset of starling syllables and USV syllables clustered together with the MNE features (1.8, and 2.4%, respectively), the majority forming a second, separate cluster. That cluster also included the sparse‐filtering features (Fig. 7).

Using the temporal and spectral projections of the modulation power spectra, we can see that the modulation power of the MNE features closely resembles the modulation power of the original syllables along both the temporal and spectral axes (Fig. 6 *C*, first column), indicating that the MNE features capture the modulations present in the syllables. Likewise, the sparse filtering features show comparable modulation projections to those of the MNE features (Fig. 6 *C*, second column). Conversely, the STC features show a narrower temporal modulation bandwidth, indicating a less accurate representation of the syllables (Fig. 6 *C*, third column).

## 4 Discussion

Our results indicate that auditory cortical neurons in the mouse, when stimulated by USVs, display multidimensional receptive fields. This result buttresses an emerging consensus that auditory cortical neurons in birds and mammals have composite receptive fields when probed with natural complex sounds. Furthermore, the results show that a natural but ethologically irrelevant heterospecific stimulus, the pitch‐shifted starling song, is an effective stimulus for the auditory cortical neurons in the mouse. Indeed, we have found that the receptive fields estimated with this stimulus have more features, both excitatory and inhibitory, than those estimated with the ethologically relevant, conspecific stimulus (USVs). However, as discussed below, the MNE features obtained with the two types of stimuli were alike, suggesting a degree of natural stimulus‐invariance in the receptive fields of these neurons. The more complex starling song may simply be more efficient at sampling the stimulus space given a limited duration of experimental recordings.

We have then taken advantage of the developments in the field of unsupervised neural networks – implicitly or explicitly based on cortical hierarchies – which can learn statistically optimal feature representations. We have trained the sparse filtering algorithm, which was used previously to learn features of the starling song (Kozlov & Gentner, 2016), to discover statistically optimal representations of these stimuli. We have compared the biological and artificial representations using a recently developed dimensionality‐reduction algorithm, UMAP, that has been previously used to capture, cluster and visualize structure of vocalizations of different animals, including songbirds and mice (Sainburg et al., 2020). One notable outcome of this analysis is that the MNE features obtained with the conspecific and heterospecific stimuli clustered together, whereas most of the stimuli and sparse‐filtering features formed a separate cluster. This implies that auditory cortical neurons in the mouse do not represent the full gamut of acoustical features present in these vocalizations. However, given the relatively low predictive power of the MNE models with these data, it would be important to investigate this question using better models of complex receptive fields, in particular models that can account for non‐stationarities and capture arbitrary stimulus‐response mappings (Keshishian et al., 2020).

In our previous work, we showed that central auditory neurons in the starling had composite receptive fields with multiple excitatory and inhibitory features (Kozlov & Gentner, 2016). We were wondering whether this receptive‐field organization was unique to songbirds, which are highly specialized vocal learners. Using the same receptive‐field analysis methods, we have now shown that neurons in the mouse auditory cortex also have composite receptive fields with a similar number of features. These similarities in the receptive‐field organization should be considered together with the similarities in the internal architecture of the auditory microcircuits in the central auditory systems of birds and mammals (Wang et al., 2010).

## Additional information

### Competing interests

The authors declare no conflicts of interest.

### Author contributions

All experimental work was performed in the lab of A.S.K. at Imperial College London. S.L.: Design of the work, acquisition, analysis and interpretation of data for the work, drafting the manuscript and revising it critically for important intellectual content. G.W.Y.A.: Design of the work, acquisition and analysis of data for the work, revising the manuscript critically. M.S.: Conception and design of the work, acquisition, analysis and interpretation of data for the work, drafting the manuscript. A.S.K: Conception and design of the work, drafting the manuscript and revising it critically for important intellectual content. All authors approved the final version of the manuscript, agree to be accountable for all aspects of the work in ensuring that questions related to the accuracy or integrity of any part of the work are appropriately investigated and resolved, and all persons designated as authors qualify for authorship, and all those who qualify for authorship are listed.

### Funding

This work was funded by Biotechnology and Biological Sciences Research Council grant ‘How do auditory cortical neurons represent ethologically relevant natural stimuli? Characterizing stimulus feature selectivity and invariance’ (BB/N008731/1).

## Supporting information


Statistical Summary Document

Peer Review History

## Data Availability

All data used for statistical comparison are included as *Supporting Information*. All other data are available upon reasonable request.
