# Supplementary material for: Composite receptive fields in the mouse auditory cortex
Source: J Physiol. 2023 Aug 14;601(18):4091–104. doi: 10.1113/JP285003 (PMC10952747; doi:10.1113/JP285003)
Supplement: Supplementary file 2 — Peer Review History [file TJP-601-4091-s002.pdf]

The Journal of Physiology

Composite receptive fields in the mouse auditory cortex

Sihao Lu, Grace W. Ang, Mark Steadman, and Andrei S. Kozlov
DOI: 10.1113/JP285003

Corresponding author(s): Andrei Kozlov (a.kozlov@imperial.ac.uk)

The referees have opted to remain anonymous.

Review Timeline:

Submission Date:	19-Oct-2021
Editorial Decision:	22-Nov-2021
Resubmission Received:	11-May-2023
Editorial Decision:	12-Jun-2023
Revision Received:	05-Jul-2023
Accepted:	12-Jul-2023

Senior Editor: Richard Carson

Reviewing Editor: Michael Okun

Transaction Report:

(Note: With the exception of the correction of typographical or spelling errors that could be a source of ambiguity, letters and reports are not edited. Depending on transfer agreements, referee reports obtained elsewhere may or may not be included in this compilation. Referee reports are anonymous unless the Referee chooses to sign their reports.)

Dear Dr Kozlov,

Re: JP-RP-2021-282507 "Composite receptive fields in the mouse auditory cortex" by Sihao Lu, Mark Steadman, Grace W. Ang, and Andrei S. Kozlov

Thank you for submitting your manuscript to The Journal of Physiology. It has been assessed by a Reviewing Editor and by 2 Referees and the reports are copied below.

Please let your co-authors know of the following editorial decision as quickly as possible.

As you will see, in its current form, the manuscript is not acceptable for publication in The Journal of Physiology. In comments to me, the Reviewing Editor expressed interest in the potential of this study, but much work still needs to be done (and this may include new experiments) in order to satisfactorily address the concerns raised in the reports.

In view of this interest, I would like to offer you the opportunity to carry out all of the changes requested in full, and to resubmit a new manuscript using the "Submit Special Case Resubmission for JP-RP-2021-282507..." on your homepage.

We cannot, of course, guarantee ultimate acceptance at this stage as the revisions required are substantial. However, we encourage you to consider the requested changes and resubmit your work to us if you are able to complete or address all changes.

A new manuscript would be renumbered and redated, but the original referees would be consulted wherever possible. An additional referee's opinion could be sought, if the Reviewing Editor felt it necessary. A full response to each of the reports should be uploaded with a new version.

I hope that the points raised in the reports will be helpful to you.

Yours sincerely,

Ian D. Forsythe
Deputy Editor-in-Chief
The Journal of Physiology
https://jp.msubmit.net
http://jp.physoc.org
The Physiological Society
Hodgkin Huxley House
30 Farringdon Lane
London, EC1R 3AW
UK
http://www.physoc.org
http://journals.physoc.org

EDITOR COMMENTS

Reviewing Editor:

Thank you for submitting your manuscript for consideration by Journal of Physiology.

Your manuscript was evaluated by two reviewers who raised several major concerns, as described in detail in their reports (please note that one of the reports was provided as a separately attached document). In particular, the reviewers found the introduction/motivation and the conclusions of the study to be rather weak. Both reviewers also question the reliance of the entire work on one method of estimating STRFs (Maximum Noise Entropy), as well as its validity and the meaning. Reviewer 1 raises several additional methodological concerns. A suggestion based on my own inspection of the manuscript is to have titles for all the figures.

Senior Editor:

There are some major issues raised by the referees, which if the authors can fully address would raise the impact of this research. If the authors are willing and able to address these concerns, JP would be pleased to receive a revised manuscript for consideration.

REFEREE COMMENTS

Referee #1:

Please see the attached review summary.

Referee #2:

This is an interesting paper that addresses an important question that is occupying many researchers in auditory neuroscience, that of characterizing the complex selectivity of cells in the higher fields of the auditory cortex. Within the motivations and assumptions of the authors, clearly spelled out in the Introduction, the study is very well planned, executed, and reported. Interestingly, the basic results "seem" to fail to reveal the hoped for selectivity of the STRFs in the mouse US cortical field to ecologically meaningful features in the USV. The STRFs were equally effective in capturing the Starling songs as the conspecific USVs, and added to the second finding that the STRFs and stimulus features artificially computed using a sparse filtering algorithm clustered apart, but overlapped regardless of the species.

To my mind, one can agree with the final conclusion of the paper that these complex STRFs are "composite" in nature. But, clearly the features found in this study are possibly primarily reflective of the algorithm (MNE). There were many other algorithms mentioned (critically for various reasons) in the introduction, and many of them would have also produced composite or composite-looking STRFs, albeit more noisy or less optimal, and not as well predictive of the responses. Nevertheless, I think it would have been valuable to the story of the authors to compare a few of these methods' STRFs to the ones extracted by the authors here. Can one obtain obviously different compositions that are more intuitive and discriminative of the species calls even if not as good predictors of the responses? If not, or if they match those found with then MNE, would strengthen strongly the conclusions of the paper that the MNE STRFs are ecologically meaningful somehow.

The other result of interest is the analysis of the USVs and starling calls to extract features that can be compared to the STRF features to determine if there was any meaningful match between the two. The negative result again suggests that perhaps one can still find a positive match if one extracts features from either stimuli or STRFs in other ways.

I realize that the analysis itself is solid and interesting, but I find the conclusion at the end somewhat contrived :

"The similar structure of auditory cortical receptive fields in the starling and in the mouse, specifically the fact that they have multiple excitatory and inhibitory subunits, and bearing in mind the ancient split between the synapsids and diapsids during amniote evolution, indicates that this property is likely a generic principle of natural sound representation".

First of all, "having multiple excitatory and inhibitory subunits", is not a profound similarity in any way, It is obvious that these higher cortical areas have such a structure as the authors point out at length in the Introduction and motivations. Second, that the similarly clustered features of the STRFs somehow reflects a generic principle of natural sound representation common to the mouse and birds is an unnecessary weak speculation.

So in short, I suggest that the authors expand on their analysis by reporting the results of 2 or 3 other (even much simpler purely linear) STRF methods and contrast then to the results of the features reported here where possible.

ADDITIONAL FORMATTING REQUIREMENTS:

-Include a Key Points list in the article itself, before the Abstract.

-Author photo and profile. First (or joint first) authors are asked to provide a short biography (no more than 100 words for one author or 150 words in total for joint first authors) and a portrait photograph. These should be uploaded and clearly labelled with the revised version of the manuscript. See Information for Authors for further details.

-You must start the Methods section with a paragraph headed Ethical Approval. A detailed explanation of journal policy and regulations on animal experimentation is given in Principles and standards for reporting animal experiments in The Journal of Physiology and Experimental Physiology by David Grundy J Physiol, 593: 2547-2549. doi:10.1113/JP270818.). A checklist outlining these requirements and detailing the information that must be provided in the paper can be found at: https://physoc.onlinelibrary.wiley.com/hub/animal-experiments. Authors should confirm in their Methods section that their experiments were carried out according to the guidelines laid down by their institution's animal welfare committee, and conform to the principles and regulations as described in the Editorial by Grundy (2015). The Methods section must contain details of the anaesthetic regime: anaesthetic used, dose and route of administration and method of killing the experimental animals.

-The Reference List must be in Journal format

-Your manuscript must include a complete Additional Information section

-Please upload separate high-quality figure files via the submission form.

-Please ensure that the Article File you upload is a Word file.

-A Statistical Summary Document, summarising the statistics presented in the manuscript, is required upon revision. It must be on the Journal's template, which can be downloaded from the link in the Statistical Summary Document section here: https://jp.msubmit.net/cgi-bin/main.plex?form_type=display_requirements#statistics

-Papers must comply with the Statistics Policy https://jp.msubmit.net/cgi-bin/main.plex?form_type=display_requirements#statistics

In summary:

-If n {less than or equal to} 30, all data points must be plotted in the figure in a way that reveals their range and distribution. A bar graph with data points overlaid, a box and whisker plot or a violin plot (preferably with data points included) are acceptable formats.

-If n > 30, then the entire raw dataset must be made available either as supporting information, or hosted on a not-for-profit repository e.g. FigShare, with access details provided in the manuscript.

-'n' clearly defined (e.g. x cells from y slices in z animals) in the Methods. Authors should be mindful of pseudoreplication.

-All relevant 'n' values must be clearly stated in the main text, figures and tables, and the Statistical Summary Document (required upon revision)

-The most appropriate summary statistic (e.g. mean or median and standard deviation) must be used. Standard Error of the Mean (SEM) alone is not permitted.

-Exact p values must be stated. Authors must not use 'greater than' or 'less than'. Exact p values must be stated to three significant figures even when 'no statistical significance' is claimed.

-Statistics Summary Document completed appropriately upon revision

-A Data Availability Statement is required for all papers reporting original data. This must be in the Additional Information section of the manuscript itself. It must have the paragraph heading "Data Availability Statement". All data supporting the results in the paper must be either: in the paper itself; uploaded as Supporting Information for Online Publication; or archived in an appropriate public repository. The statement needs to describe the availability or the absence of shared data. Authors must include in their Statement: a link to the repository they have used, or a statement that it is available as Supporting Information; reference the data in the appropriate sections(s) of their manuscript; and cite the data they have shared in the References section. Whenever possible the scripts and other artefacts used to generate the analyses presented in the paper should also be publicly archived. If sharing data compromises ethical standards or legal requirements then authors are not expected to share it, but must note this in their Statement. For more information, see our Statistics Policy.

-Please include an Abstract Figure. The Abstract Figure is a piece of artwork designed to give readers an immediate understanding of the research and should summarise the main conclusions. If possible, the image should be easily 'readable' from left to right or top to bottom. It should show the physiological relevance of the manuscript so readers can assess the importance and content of its findings. Abstract Figures should not merely recapitulate other figures in the manuscript. Please try to keep the diagram as simple as possible and without superfluous information that may distract from the main conclusion(s). Abstract Figures must be provided by authors no later than the revised manuscript stage and should be uploaded as a separate file during online submission labelled as File Type 'Abstract Figure'. Please ensure that you include the figure legend in the main article file. All Abstract Figures should be created using BioRender. Authors should use The Journal's premium BioRender account to export high-resolution images. Details on how to use and access the premium account are included as part of this email.

Confidential Review **19-Oct-2021**

Review of "Composite receptive fields in the mouse auditory cortex"

Summary

The activity of auditory or visual cortical neurons is frequently interpreted as a computation on the sensory input. A leading approach is to determine the types of stimuli eliciting larger neuronal responses. Often, the stimulus space is thought to follow simple geometry and a neuron's *receptive field*, or *tuning curve* is defined as the region of the sensory stimulus space where the neuron's response modulates.

Here, Lu et al. highlight two issues complicating the study of sensory neuron responses: 1) neurons responding to multiple dimensions of the stimulus space, and 2) neurons exhibiting different computations, i.e. estimated stimulus$\leftrightarrow$response tuning, when probed with distinct sets of stimuli. The authors claim that for these two reasons, past work probing the response of mouse auditory cortex neurons using artificial stimuli cannot determine the processing of natural stimuli. Further, the authors mention works describing responses to natural auditory stimuli in other mammals (and birds) but suggest that doing so in mice is important since mice offer a powerful model organism in neuroscience research.

To address these issues, the authors recorded activity of neurons in the primary auditory cortex of anaesthetized mice responding to curated sets of mice ultrasonic vocalisations (USVs) as well as to starling songs pitch-adjusted to the USVs' ultrasonic range. Using an analysis method capable of extracting multiple features eliciting or inhibiting neural response the authors describe the dimensionality of the response, compare responses to USVs and pitch-shifted starling song, and compare the natural stimulus features contributing to the neurons' responses with features defining the stimulus space as extracted by a neural network algorithm.

Addressing the complexity of auditory neuronal responses in mice is a direction worth pursuing. A strength of the work by Lu et al. is the careful preparation of stimulus datasets and the acquisition of physiological recordings. These form a solid scientific basis with which the authors can address questions about neural encoding in mouse auditory cortex. However, major weaknesses - in framing the work and in the analysis carried - greatly reduce the quality of the manuscript. These weaknesses are detailed below as *Major Concerns* and will require an extensive revision to address properly - either as a revised submission or following a rejection with an option to resubmit. At this point I refrain from detailing minor concerns since the manuscript requires extensive work on major concerns.

Major concerns

Background, framing, and citations

The manuscript does not provide the background required for the line of inquiry it presents in two main aspects:
Providing the background needed and leading to the research hypothesis or question: Much general language and argumentation is devoted to justifying the main analysis method used (MNE). This is an irrelevant introduction to the research question and the result presented. If, for example, the paper was dealing with differences in receptive field properties estimated with spike-triggered averages, covariance, or other methods introduced (but not used) then it would make sense to introduce them. Otherwise, the method used needs describing in the Methods section and discussion of other potential methods, if the authors deem it needed, can be part of the Discussion section.

The authors provide very little justification to their research questions and work. First the authors mention composite receptive fields already identified using artificial (e.g. pure tones) stimuli. The authors do not mention in which animals these discoveries were made. Later, exactly one part of one sentence at the end of the introduction mentions the strength of mice as a model animal - suggesting that past work was done in mice. The authors also mention work in other animal models, birds and mammals, identifying composite receptive fields using natural stimuli. From these two points the reader may construe that there is no past work identifying composite receptive fields in mouse auditory cortex using natural stimuli. This should be the case because this is what motivates the presented research but it needs to be clearly stated and supported by a comprehensive introduction of what was done in mice.

Assuming that the presented work is indeed the first characterization of mouse auditory cortex responses to natural stimuli showing composite receptive fields, it would present an advance to the field - albeit a small one. The comments below suggest potential avenues of strengthening the contribution.
Providing the background and citations needed to correspond with previous work: The manuscript presents very little referencing of past work. In fact, major labs working on mouse auditory cortex are ignored as well as their findings. This must be remedied for the manuscript to be considered for publication. The Journal of Physiology addresses a wide readership of researchers and students. A serious introduction to the field of auditory cortex processing, especially in mice, is a must.

Additionally, many claims in the manuscript require either explanation or a citation, not just being mentioned in passing. When revising their manuscript, the authors should ask a colleague to read it and highlight all such cases. In this review, it is not useful to list all such points in the text since the manuscript needs much change.

Methods and Analysis

Several major gaps exist in ascertaining the adequacy of methods and analyses for answering research questions in the manuscript:

Adequacy of MNE model fits: The authors do not provide goodness-of-fit measures showing how well the MNE model describe the firing properties of the neurons they recorded. The discussion mentions that the MNE models poorly fitted the data. At this point it is not clear if the results can be trusted.

The authors could potentially use simulated neuronal responses (sampled from models with added noise yielding similar degree of misalignment between models and data) to support their ability to extract the correct features. The authors can also use likelihood-based measures as the MNE model is a probabilistic one, or use the KS goodness-of-fit measure.

The authors do use a correlation measure between model prediction and spike trains. If this translates to goodness-of-fit or explained variance measures then the authors can explain the adequacy in the Methods section.

Using pitch-shifted birdsong: The authors suggest that using pitch-shifted starling song is ethological. There is no basis provided for this claim, which is in the heart of the manuscript. The MNE models for neural responses to birdsong were different than those fitted to responses to USVs. The authors should do a better comparison, using the responses of each neuron to both stimulus sets in a paired manner. Since the composite receptive fields when using birdsong were different than those for USVs (different number of features) it is not clear if birdsong is better in any way than just playing tones. The authors should make a very careful comparison supporting their claims. Otherwise, the paper needs re-framing - either removing the starling data or treating it as you would pure tones - an unnatural sound.

Probing the dimensionality of responses:

- The authors need to make sure the stimulus sets they prepared are adequate for probing the dimensionality of responses. In other words, it is not clear what ranges of dimensonality can be found by the method used. This becomes clear when observing that larger quantities of stimuli result in increased dimensionality of responses. The authors should clearly show how their results are sensitive to the choice of natural USV datasets (size, content, intrinsic dimensions)

- (minor) when comparing quantities of observed features the authors used the two-sample Kolmogorov-Smirnov test. This test checks for equality of distributions and will declare significant difference also when the mean or median do not change. (e.g. if the variance changes). The authors should choose a test matching the claims they make.

Probing features of responses:

- The authors provide very little detail and analysis about what the extracted features may actually mean, how they depend on the choices made in preparing the stimuli set?

- The authors should compare the features they find when using USVs, starling song, as well as 'traditional' tone and amplitude modulation (as in works by T. Sharpee).

- The authors use UMAP to compare extracted features. It is not clear why this is an adequate way to compare? It is not clear why noise was added to this process? How does adding noise affect the results?

- The authors do not quantify claims of similarity using the UMAP. They just claim it. What sets of features will NOT cluster in this analysis?

- The figure showing the results of the UMAP embedding is used to make claims about similarity (with no quantification). The work by Sainburg et al (2020) showed a much greater separability of vocalization classes than the 3 clusters presented here. This is strange and inexplicable. It calls the method into questioning. The authors must clearly explain their choice of analysis, how it meets their research question, and how they quantify, validate, and interpret the result. The current text lacks all of these.

Recording location: The authors should provide evidence ascertaining that the neurons they recorded were indeed in the primary auditory cortex.

Response letter

Dear Editors,

We are grateful for your consideration of our manuscript, *JP-RP-2021-282507 "Composite receptive fields in the mouse auditory cortex"*. We would also like to extend our gratitude to the reviewers for their valuable feedback regarding the manuscript. We have taken their comments into account, performed additional experiments and data analyses, and have updated the manuscript accordingly. Below we have copied the comments by the editors and reviewers and included our responses for each point.

Editor 1

Editor Point 1.1— *Thank you for submitting your manuscript for consideration by Journal of Physiology.*

Your manuscript was evaluated by two reviewers who raised several major concerns, as described in detail in their reports (please note that one of the reports was provided as a separately attached document). In particular, the reviewers found the introduction/motivation and the conclusions of the study to be rather weak. Both reviewers also question the reliance of the entire work on one method of estimating STRFs (Maximum Noise Entropy), as well as its validity and the meaning. Reviewer 1 raises several additional methodological concerns. A suggestion based on my own inspection of the manuscript is to have titles for all the figures.

Reply: Thank you for considering our manuscript for publication in the Journal of Physiology. We appreciate the thorough evaluations performed by the reviewers and have taken their comments and suggestions into consideration in revising our manuscript. In particular, we have strengthened the introduction and conclusion, and have added clarity to our reliance on the Maximum Noise Entropy method for estimating STRFs. We have also addressed the additional methodological concerns raised by Reviewer 1. Additionally, we have added figure titles to help clarify the results presented in the figures. We hope that our revisions have addressed the major concerns raised by the reviewers and that our manuscript will be given favorable consideration for publication.

Editor 2

Editor Point 2.1— *There are some major issues raised by the referees, which if the authors can fully address would raise the impact of this research. If the authors are willing and able to address these concerns, JP would be pleased to receive a revised manuscript for consideration.*

Reply: We thank you for your feedback and the opportunity to revise our manuscript. We have taken the points raised by the reviewers into consideration and have addressed them to the best of our abilities. We hope that our revised manuscript accurately reflects the improvements we have made and that it will be well received by Journal of Physiology.

Reviewer 1

Reviewer Point 1.1— ***Summary:*** *The activity of auditory or visual cortical neurons is frequently interpreted as a computation on the sensory input. A leading approach is to determine the types of stimuli eliciting larger neuronal responses. Often, the stimulus space is thought to follow simple geometry and a neuron's receptive field, or tuning curve is defined as the region of the sensory stimulus space where the neuron's response modulates. Here, Lu et al. highlight two issues complicating the study of sensory neuron responses: 1) neurons responding to multiple dimensions of the stimulus space, and 2) neurons exhibiting different computations, i.e. estimated stimulus↔response tuning, when probed with distinct sets of stimuli. The authors claim that for these two reasons, past work probing the response of mouse auditory cortex neurons using artificial stimuli cannot determine the processing of natural stimuli. Further, the authors mention works describing responses to natural auditory stimuli in other mammals (and birds) but suggest that doing so in mice is important since mice offer a powerful model organism in neuroscience research. To address these issues, the authors recorded activity of neurons in the primary auditory cortex of anaesthetized mice responding to curated sets of mice ultrasonic vocalisations (USVs) as well as to starling songs pitch-adjusted to the USVs' ultrasonic range. Using an analysis method capable of extracting multiple features eliciting or inhibiting neural response the authors describe the dimensionality of the response, compare responses to USVs and pitch-shifted starling song, and compare the natural stimulus features contributing to the neurons' responses with features defining the stimulus space as extracted by a neural network algorithm. Addressing the complexity of auditory neuronal responses in mice is a direction worth pursuing. A strength of the work by Lu et al. is the careful preparation of stimulus datasets and the acquisition of physiological recordings. These form a solid scientific basis with which the authors can address questions about neural encoding in mouse auditory cortex. However, major weaknesses - in framing the work and in the analysis carried - greatly reduce the quality of the manuscript. These weaknesses are detailed below as Major Concerns and will require an extensive revision to address properly - either as a revised submission or following a rejection with an option to resubmit. At this point I refrain from detailing minor concerns since the manuscript requires extensive work on major concerns.*

Reply: Thank you for your comprehensive feedback on our manuscript. We understand that major weaknesses in the framing and analysis of our work were identified and we are committed to making the necessary revisions to improve the manuscript. We appreciate the reviewer's recognition of the careful preparation of our stimulus datasets and physiological recordings. We will work to address the major concerns raised by the reviewer and will revise the manuscript accordingly. We appreciate the opportunity to resubmit a stronger and more comprehensive paper.

Reviewer Point 1.2— *Providing the background needed and leading to the research hypothesis or question: Much general language and argumentation is devoted to justifying the main analysis method used (MNE). This is an irrelevant introduction to the research question and the result presented. If, for example, the paper was dealing with differences in receptive field properties estimated with spike-triggered averages, covariance, or other methods introduced (but not used) then it would make sense to introduce them. Otherwise, the method used needs describing in the Methods section and discussion of other potential methods, if the authors deem it needed, can be part of the Discussion section.*

Reply: We understand your point of view and appreciate the suggestion to move the argumentation of using Maximum Noise Entropy (MNE) to the Methods section. However, our aim was to provide the background and context for why the MNE method was chosen for this study. The major reason that receptive fields have not been estimated from natural stimuli is due to the computational restrictions of previous methods, which have been overcome with the MNE method. We believe that this background information is important for the reader to fully understand the rationale behind our choice of method and why other methods were not suitable for this purpose.

Reviewer Point 1.3— *The authors provide very little justification to their research questions and work. First the authors mention composite receptive fields already identified using artificial (e.g. pure tones) stimuli. The authors do not mention in which animals these discoveries were made. Later, exactly one part of one sentence at the end of the introduction mentions the strength of mice as a model animal - suggesting that past work was done in mice. The authors also mention work in other animal models, birds and mammals, identifying composite receptive fields using natural stimuli. From these two points the reader may construe that there is no past work identifying composite receptive fields in mouse auditory cortex using natural stimuli. This should be the case because this is what motivates the presented research but it needs to be clearly stated and supported by a comprehensive introduction of what was done in mice. Assuming that the presented work is indeed the first characterization of mouse auditory cortex responses to natural stimuli showing composite receptive fields, it would present an advance to the field - albeit a small one. The comments below suggest potential avenues of strengthening the contribution.*

Reply: Thank you for pointing out the lack of justification in our research questions and work. We have revised the introduction to clearly state that our research is the first characterization of composite receptive fields in mouse auditory cortex using natural stimuli. We have also provided more context and information about previous work in this area to give a clearer understanding of the significance of our research (detailed in the reply to the following point).

Reviewer Point 1.4— *Providing the background and citations needed to correspond with previous work: The manuscript presents very little referencing of past work. In fact, major labs working on mouse auditory cortex are ignored as well as their findings. This must be remedied for the manuscript to be considered for publication. The Journal of Physiology addresses a wide readership of researchers and students. A serious introduction to the field of auditory cortex processing, especially in mice, is a must. Additionally, many claims in the manuscript require either explanation or a citation, not just being mentioned in passing. When revising their manuscript, the authors should ask a colleague to read it and highlight all such cases. In this review, it is not useful to list all such points in the text since the manuscript needs much change.*

Reply: We appreciate the reviewer's feedback and we have made a thorough revision to include references to relevant earlier work in the field of auditory cortex processing. This includes references to studies on frequency selectivity in the inferior colliculus [Aitkin et al., 1975, Aitkin and Moore, 1975] and along the central auditory pathway [Calford et al., 1983] of cats, characterising single-unit responses using carefully designed artificial stimuli in ferrets [Kowalski et al., 1996a,b, Depireux et al., 2001], feature processing in auditory cortex of primates [deCharms et al., 1998], the use of stimuli with natural statistics in rats [Machens et al., 2004], and stimulation with conspecific vocalizations in songbirds [Doupe, 1997, Theunissen et al., 2000, Grace et al., 2003], ferrets [Harper et al., 2016] and guinea pigs

[Laudanski et al., 2012]. With regards to work on mouse auditory cortex specifically, we cite early work on tonotopy in mouse cortex [Stiebler et al., 1997], more recent studies on spectro-temporal structure of receptive fields [Linden et al., 2003] and on cell-type specific receptive field properties [Li et al., 2015]. These references provide a comprehensive background and demonstrate our effort to accurately reference past work in the field. We believe that these additions will greatly enhance the reader's understanding of our research.

Reviewer Point 1.5— *Adequacy of MNE model fits: The authors do not provide goodness-of-fit measures showing how well the MNE model describe the firing properties of the neurons they recorded. The discussion mentions that the MNE models poorly fitted the data. At this point it is not clear if the results can be trusted. The authors could potentially use simulated neuronal responses (sampled from models with added noise yielding similar degree of misalignment between models and data) to support their ability to extract the correct features. The authors can also use likelihood-based measures as the MNE model is a probabilistic one, or use the KS goodness-of-fit measure. The authors do use a correlation measure between model prediction and spike trains. If this translates to goodness-of-fit or explained variance measures then the authors can explain the adequacy in the Methods section.*

Reply: We acknowledge the reviewer's concerns regarding the adequacy of the MNE model fits and the lack of clarity regarding the use of the correlation coefficient to show goodness-of-fit. Our discussion indeed mentions that the MNE models poorly fitted the data, raising concerns about the reliability of the results. We appreciate the reviewer's suggestion to use simulated neuronal responses or likelihood-based measures, as well as the KS goodness-of-fit measure. We hope to clarify our reasoning in the response below.

The rationale behind our choice of the correlation coefficient is that it is a commonly used goodness-of-fit measure to determine how well the receptive field model captures the tuning of a neuron [Theunissen et al., 2000, Woolley et al., 2006, Calabrese et al., 2011, Laudanski et al., 2012]. David and Gallant [2005] provide an interpretation of the (squared) correlation coefficient and show that it is equivalent to the variance explained in noiseless conditions.

Since neural responses to repeated presentations of identical stimuli are inherently variable, model evaluation should take into account this source of neuronal variability which cannot be explained by fitting the model. In our manuscript, we normalized correlation to neural variability.

Other studies have reported correlation coefficients in a similar range to ours, using different receptive field estimation methods. Laudanski et al. [2012] reported a mean correlation of 0.29 for receptive fields derived from guinea pig auditory responses to vocalizations. Calabrese et al. [2011] reported correlations values of 0.47 for receptive fields estimated using GLM and noted lower correlation (0.36) for neurons with low firing rates.

The MNE model was previously evaluated using simulated visual neurons responding to natural images [Fitzgerald et al., 2011], and shown to be able to recover features from simulated cells. In addition, several studies have been published which show receptive field features estimated using this method [Kozlov and Gentner, 2016, Atencio and Sharpee, 2017, Rowekamp and Sharpee, 2017].

Reviewer Point 1.6— *Using pitch-shifted birdsong: The authors suggest that using pitch-shifted starling song is ethological. There is no basis provided for this claim, which is in the heart of the manuscript. The MNE models for neural responses to birdsong were different than those fitted to responses to USVs. The authors should do a better comparison, using the responses of each neuron*

to both stimulus sets in a paired manner. Since the composite receptive fields when using birdsong were different than those for USVs (different number of features) it is not clear if birdsong is better in any way than just playing tones. The authors should make a very careful comparison supporting their claims. Otherwise, the paper needs re-framing - either removing the starling data or treating it as you would pure tones - an unnatural sound.

Reply: We would like to thank the reviewer for bringing up the issue regarding our claim about the use of pitch-shifted starling song as a natural stimulus. We apologize if our initial explanation was unclear and would like to provide further clarification.

In our study, we stated that pitch-shifted starling song was an ethologically *irrelevant* stimulus, but we would like to clarify that it is nevertheless a complex stimulus with natural statistics. Hence the starling song was chosen as a stimulus for comparison, instead of tones which were simple and unnatural stimuli, to investigate whether the ethological relevance (USVs vs. starling song) influenced the receptive fields.

We studied the generalization ability of MNE models trained on USVs and starling songs by using models trained on one to predict responses to the other. The predictive power decreased significantly when the model predicted responses to the other stimulus type, especially for models trained on pitch-shifted starling songs as their cross-stimulus response prediction was worse than that of the models trained on USVs (Fig. 1; $p < 0.001$).

Other studies [Calabrese et al., 2011, Woolley et al., 2006, Laudanski et al., 2012] have noted poor across-stimulus class generalization, due to nonlinear response properties, which our results support as well.

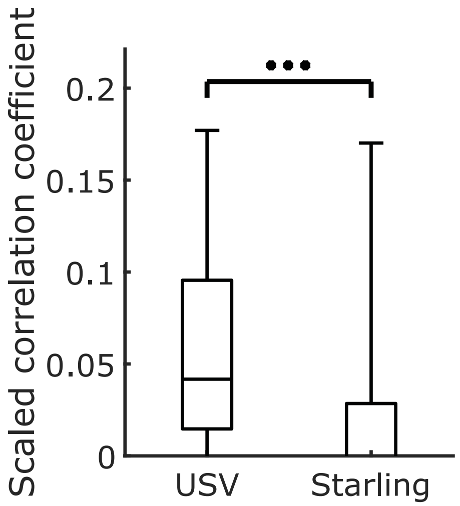

Figure 1: Prediction accuracy across stimulus types given by correlation coefficient of predicted neural activity to other stimulus type. *USV* label denotes models trained with responses to USV stimuli predicting responses to starling songs, and *Starling* label denotes models trained starling song responses predicting responses to USVs. ***$p < 0.001$ (paired t-test).

Reviewer Point 1.7— *Probing the dimensionality of responses: The authors need to make sure the stimulus sets they prepared are adequate for probing the dimensionality of responses. In other*

words, it is not clear what ranges of dimensionality can be found by the method used. This becomes clear when observing that larger quantities of stimuli result in increased dimensionality of responses. The authors should clearly show how their results are sensitive to the choice of natural USV datasets (size, content, intrinsic dimensions)

Reply: We extend our thanks to the reviewer for raising concerns regarding the adequacy of the stimulus sets prepared for probing the dimensionality of responses. We agree that the results could be sensitive to the size, content and intrinsic dimensionality of the stimulus. In response, we provide further analysis which we hope will alleviate some of the reviewer's concerns.

The MNE uncovers multiple dimensions in the stimulus space which modulate the neuron's response; the number of features estimated by the model reflects the dimensionality of response to the stimulus. To investigate the effect of the length of stimulus used, we trained the MNE models on increasing subsets of the original stimulus, ranging from 50% to 100%.

For the models trained on USV data, using 100% of the training dataset increased model prediction accuracy relative to 50% (Fig. 2A; $p < 0.001$, Kruskall-Wallis test with Bonferroni multiple comparison correction). However, this improvement in performance is already non-significant when using 60% of our training data compared to 100% ($p = 0.07$, Kruskall-Wallis test with Bonferroni multiple comparison correction). For models trained on starling song, we observe that there is no significant difference in performance between the different subsets used ($p = 0.98$, Kruskall-Wallis test).

The number of estimated excitatory features similarly is stable with respect to fraction of training data used (Fig. 2B; $p = 0.07$ and $p = 0.99$ for USV and starling song respectively). In contrast the number of significant features for the USV trained models only stabilizes after 70% of the data (Fig. 2C; $p = 0.23$, Kruskall-Wallis test with Bonferroni multiple comparison correction) and the number of significant features for the starling models remains stable for all subset percentages ($p = 0.98$, Kruskall-Wallis test)

Based on these findings, it appears that the size of the dataset used does not have a substantial impact on the results of our study. However, we acknowledge the importance of further investigating the sensitivity of our results to the choice of natural USV datasets and will remain cautious in our interpretation of these findings.

Reviewer Point 1.8— *(minor) when comparing quantities of observed features the authors used the two-sample Kolmogorov-Smirnov test. This test checks for equality of distributions and will declare significant difference also when the mean or median do not change. (e.g. if the variance changes). The authors should choose a test matching the claims they make.*

Reply: We are grateful to the reviewer for pointing out this statistical oversight in our analyses. We have used the Wilcoxon rank sum test instead of the two-sample Kolmogorov-Smirnov test when comparing the number of features observed. This test compares the median of two independent samples. The captions of Figures 3 and 5 have been updated to reflect this change. Likewise the text in the main body of the manuscript has been corrected. In addition, we have applied a Bonferroni-Holm correction for multiple comparisons when testing for statistical differences between numbers of excitatory and inhibitory features of USV and starling song trained models.

Reviewer Point 1.9— *Probing features of responses: The authors provide very little detail and analysis about what the extracted features may actually mean, how they depend on the choices made in preparing the stimuli set?*

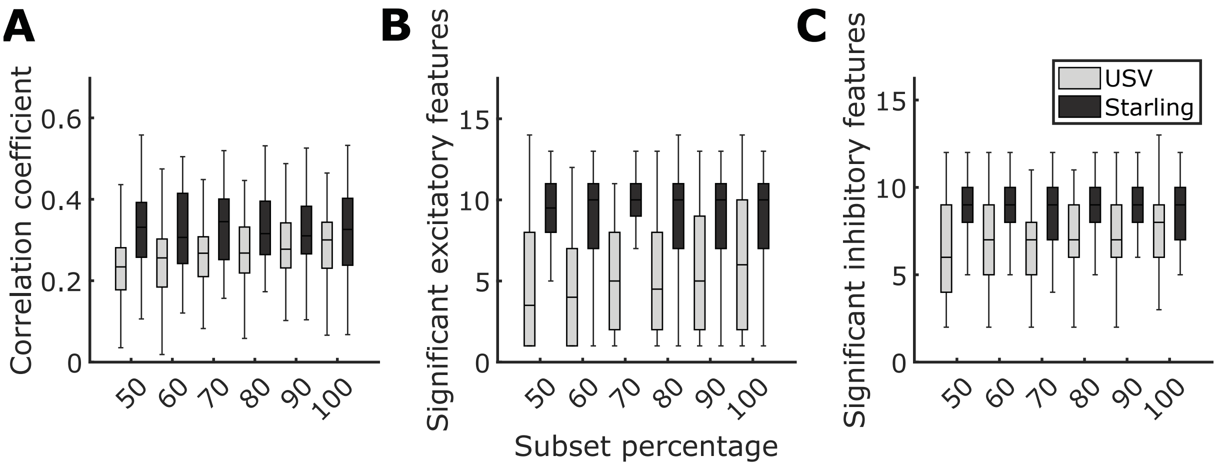

Figure 2: **(A)** Model performance as given by correlation coefficient between real and predicted neural activity of MNE models trained on either USVs or pitch-shifted starling songs. The performance is reported for different percentages of the training data used in the original manuscript.**(B)**, **(C)** The number of excitatory and inhibitory features estimated from the MNE models trained on either USVs or pitch-shifted starling songs, relative to the subset percentage.

Reply: The reviewer's observation regarding the level of detail and analysis about the extracted features is well taken. We agree that our initial manuscript could have provided more detail and analysis about the extracted features.

To address this, we performed additional analyses to characterize the acoustical properties of the extracted features, we calculated the modulation power spectrum [Singh and Theunissen, 2003] which shows signal power as a function of temporal modulations and spectral modulations. These results have been added to Figure 6 of the original manuscript (Fig. 6C here). We can see that the modulation power of the MNE features closely resembles the modulation power of the original syllables along both the temporal and spectral axes (Fig. 6C, first column), indicating that the MNE features capture the modulations present in the syllables. Likewise, the sparse filtering features show comparable modulation projections to those of the MNE features (Fig. 6C, second column). Conversely, the STC features show a narrower temporal modulation bandwidth, indicating a less accurate representation of the syllables (Fig. 6C, third column).

Reviewer Point 1.10— *The authors should compare the features they find when using USVs, starling song, as well as 'traditional' tone and amplitude modulation (as in works by T. Sharpee).*

Reply:

We appreciate the suggestion from the reviewer to extend our analyses to features obtained with traditional tone and amplitude modulated stimuli. Thus we have now performed additional experiments where we present both USVs and artificial melody composed of tones to the neurons to compare receptive fields to complex and simple stimuli (n = 34). The artificial melody was a randomized sequence of 26 tones, linearly spaced between 30 kHz and 90 kHz. Each tone was 200ms in duration and occurred 5 times in the sequence. MNE features for responses to tones were estimated using a stimulus representation with 32 spectral bins by 12 timesteps. Compared to MNE fits to USV responses,

correlation values were higher when the MNE models were fit to responses to tones (Fig. 3; CC_{usv} : $0.32; CC_{tones} : 0.42; p < 0.01$; Wilcoxon rank sum test). Stimulating cells with USVs produced more

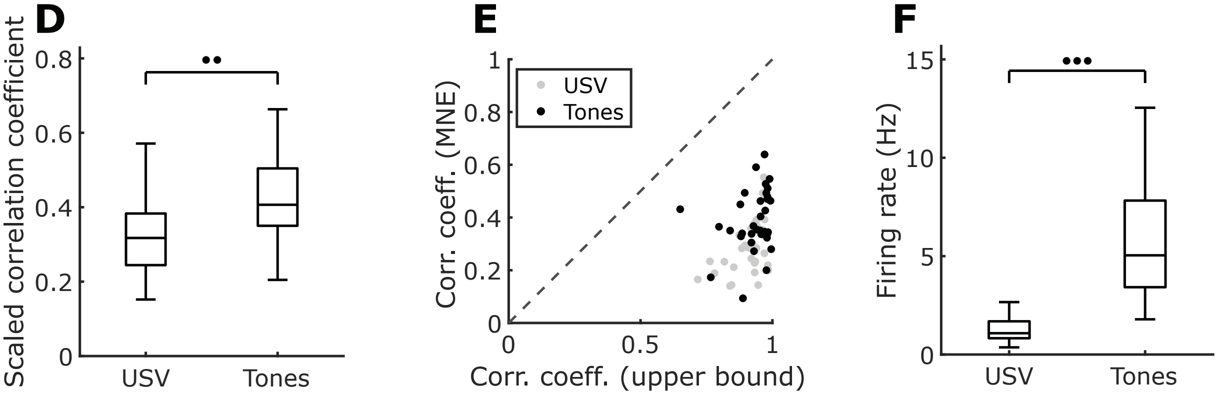

Figure 3: **(D)** Correlation coefficients between MNE model predictions and recorded neural activity in response to stimulation with either USVs ($n = 34$, $\mu = 0.32$, $\sigma = 0.10$) or pure tones ($n = 34$, $\mu = 0.42$, $\sigma = 0.13$). $**P < 0.01$ (Wilcoxon rank sum test). The correlation coefficients are scaled by the correlation coefficient between the responses to repeated presentations of the same stimulus in the test set. **(E)** Same as (B), but data shown are for predictions based on models trained on USVs (grey) or pure tones (black). **(F)** Comparison of the firing rates of the recorded units in response to either USVs ($n = 34$, $\mu = 1.4$ Hz, $\sigma = 1.2$ Hz) or pure tones ($n = 34$, $\mu = 6.5$ Hz, $\sigma = 5.2$ Hz). $***P < 0.001$ (Wilcoxon rank sum test). In both cases, the firing rates were calculated using full recordings and not only test data.

excitatory features compared to stimulation with pure tones (Fig.4; $p < 0.001$), showing that the dimensionality of responses depended on whether simple or complex stimuli were used to probe the receptive field of the cell.

Reviewer Point 1.11— *The authors use UMAP to compare extracted features. It is not clear why this is an adequate way to compare? It is not clear why noise was added to this process? How does adding noise affect the results? The authors do not quantify claims of similarity using the UMAP. They just claim it. What sets of features will NOT cluster in this analysis? The figure showing the results of the UMAP embedding is used to make claims about similarity (with no quantification). The work by Sainburg et al (2020) showed a much greater separability of vocalization classes than the 3 clusters presented here. This is strange and inexplicable. It calls the method into questioning. The authors must clearly explain their choice of analysis, how it meets their research question, and how they quantify, validate, and interpret the result. The current text lacks all of these.*

Reply: Thank you for raising this important question about the use of UMAP in our analysis. We apologize for not being more clear about the methodology and results of our analysis. UMAP, a nonlinear dimensionality reduction algorithm, previously showed clustering results that captured complex features of animal vocalizations in interpretable ways, and showed good correspondence with experimental labels [Sainburg et al., 2020].

In the work by Sainburg et al. [2020], each UMAP space contained projections of the same type of data representation - spectrograms of syllables (discrete elements segmented from vocalization wave-

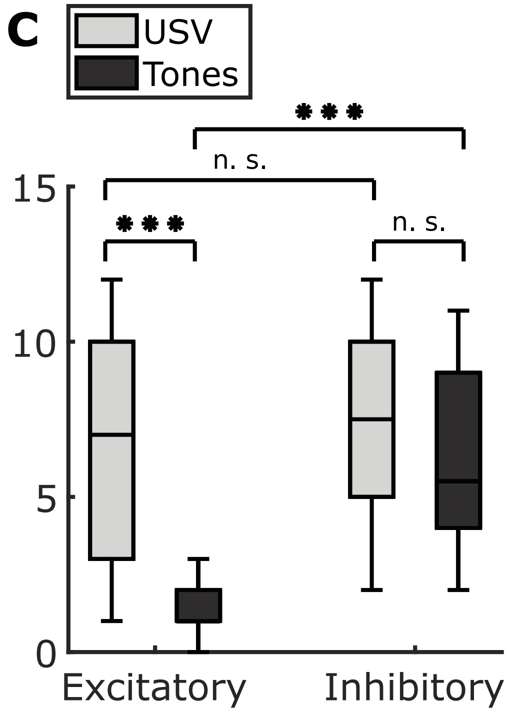

Figure 4: **(C)** Number of excitatory ($\mu = 6.44, \sigma = 3.3$) and inhibitory ($\mu = 7.29, \sigma = 2.68$) features when the MNE model is trained with responses to USVs versus number of excitatory ($\mu = 1.38, \sigma = 0.99$) and inhibitory ($\mu = 6.18, \sigma = 2.99$) features of models trained on responses to pure tones ($n = 34$). $***P < 0.001$ (Wilcoxon rank sum test, Bonferroni-Holm multiple comparison correction).

forms). The authors found good discriminability in features such as individual identity, species identity and geographic variability within each animal type. Figure 6 of our manuscript instead shows receptive field features estimated with different methods and two types of animal vocalizations projected into the same UMAP space. Our intention was to compare (i) neuronal receptive fields across con- and heterospecific vocalizations and (ii) neuronal representations extracted from experimental recordings versus learnt features from the sparse filtering algorithm.

We applied UMAP as we wanted to compare representations learnt by an unsupervised feature learning algorithm (sparse filtering) to those of experimentally recorded units (which were extracted by MNE). Our previous work [Kozlov and Gentner, 2016] showed that receptive fields of starling NCM neurons and sparse filtering features display similarities in two defined properties of the receptive field, namely the temporal and spectral modulation. Using this dimensionality reduction method, we wanted to investigate any similarity between the features that are not readily apparent by comparing specific properties. Hence we projected the two types of optimized representations (neuronal MNE features and sparse filtering features) together with the original input (spectrograms of vocalization syllables) into the same UMAP latent space to visualize any overlaps. As a control, spectrograms of white noise were also projected into the UMAP space - these were not expected to cluster with the other features and indeed they did not.

We have added a clustering analysis based on the HDBSCAN algorithm [McInnes and Healy, 2017] which has been shown to reliably extract clusters in UMAP embeddings. In Fig. 5 we can see that four clusters are clearly delineated. These four clusters consist of one cluster which contains all the noise features, one cluster which contains the individual syllables in combination with the sparse filtering features, one cluster containing all the MNE features and a final cluster containing the STC features.

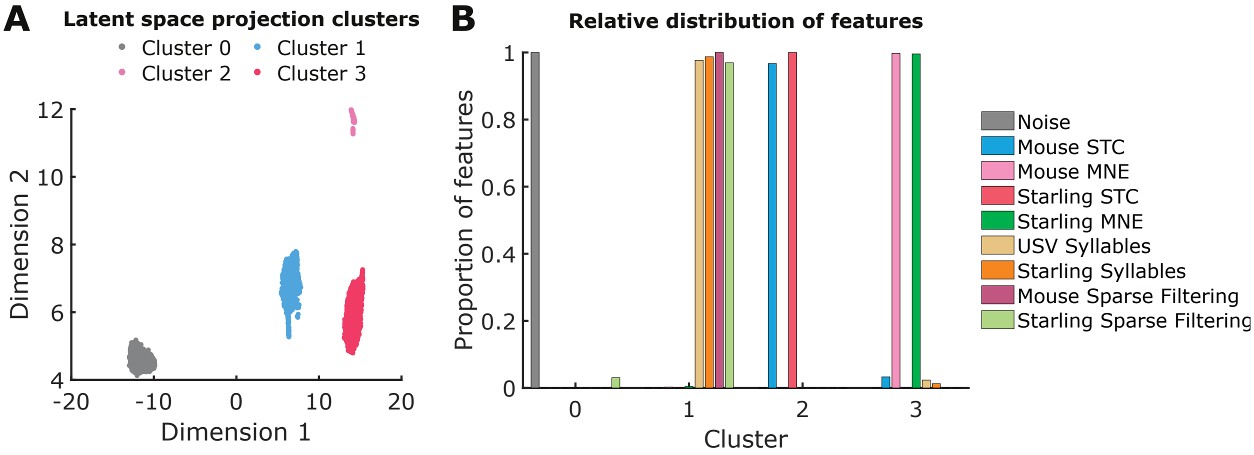

Figure 5: *Clustering and distribution analysis of features.* **(A)** Clustering UMAP projections of: segmented syllables present in USVs and pitch-shifted starling songs, significant features estimated using both the STC method and the MNE models, features estimated using the sparse filtering method, and randomly generated noise. Four clusters were detected and are labeled. Cluster 0 clearly separates the noise from the other features, whilst cluster 1 includes the USVs and starling-song syllables segmented from the stimuli and the sparse filtering features estimated from the stimuli. Lastly, both the STC and the MNE features are separated into their own respective clusters. (**B**) Relative distribution of each class to the four detected clusters.

We must note also that the resolution of the spectra used by Sainburg et al. [2020] is much higher than the resolution used for our analyses, allowing a more fine-grained delineation between the vocalization classes. Our choice of resolution is constrained by the number of model parameters we can fit using data recorded within a single experimental session, whereas this constraint did not apply to the work of Sainburg et al. [2020] as they focused on acoustics but not on neuronal responses.

Reviewer Point 1.12— *Recording location: The authors should provide evidence ascertaining that the neurons they recorded were indeed in the primary auditory cortex.*

Reply: In addition to carefully measuring the coordinates when performing the craniotomy, we monitored the local field potential (LFP) as we lowered the electrode into the brain tissue. During this process, we played repeated noise bursts to the animal, observing stimulus-locked responses. This provides evidence of our recordings being made in the primary auditory cortex.

Reviewer 2

Reviewer Point 2.1— *This is an interesting paper that addresses an important question that is occupying many researchers in auditory neuroscience, that of characterizing the complex selectivity of cells in the higher fields of the auditory cortex. Within the motivations and assumptions of the authors, clearly spelled out in the Introduction, the study is very well planned, executed, and reported. Interestingly, the basic results "seem" to fail to reveal the hoped for selectivity of the STRFs in the mouse US cortical field to ecologically meaningful features in the USV. The STRFs were equally effective in capturing the Starling songs as the conspecific USVs, and added to the second finding that the STRFs and stimulus features artificially computed using a sparse filtering algorithm clustered apart, but overlapped regardless of the species.*

Reply: We thank the reviewer for their positive comments regarding the work described in the manuscript. We have performed additional analyses to further compare the difference in features extracted using the two sets of stimuli. We show that models trained on the USVs perform better at predicting responses of cells presented with starling songs than vice versa (Fig. 1). This implies that the features estimated using the USV stimuli have improved generalization capabilities.

Reviewer Point 2.2— *To my mind, one can agree with the final conclusion of the paper that these complex STRFs are "composite" in nature. But, clearly the features found in this study are possibly primarily reflective of the algorithm (MNE). There were many other algorithms mentioned (critically for various reasons) in the introduction, and many of them would have also produced composite or composite-looking STRFs, albeit more noisy or less optimal, and not as well predictive of the responses. Nevertheless, I think it would have been valuable to the story of the authors to compare a few of these methods' STRFs to the ones extracted by the authors here. Can one obtain obviously different compositions that are more intuitive and discriminative of the species calls even if not as good predictors of the responses? If not, or if they match those found with then MNE, would strengthen strongly the conclusions of the paper that the MNE STRFs are ecologically meaningful somehow.*

Reply: We thank the reviewer for acknowledging the conclusion of our paper that the complex STRFs are "composite" in nature and for suggesting a valuable comparison with other methods. In response to this suggestion, we have estimated receptive field features of the recorded units using spike-triggered covariance (STC) and included these features into the UMAP projection (Fig. 6). Notably, they form their own cluster, suggesting that they are distinct from features estimated using MNE. Concurrently, they are separate from the cluster formed by vocalizations, implying that they are not more intuitive or discriminative of the species' calls (Fig. 5). Additionally, we find that models using the features uncovered by STC are worse predictors of the neural activity (7).

Reviewer Point 2.3— *The other result of interest is the analysis of the USVs and starling calls to extract features that can be compared to the STRF features to determine if there was any meaningful match between the two. The negative result again suggests that perhaps one can still find a positive match if one extracts features from either stimuli or STRFs in other ways.*

Reply: The lack of overlap in UMAP space between syllable spectrograms and MNE features could reflect differences in data representation. The MNE STRF features are a low-dimensional subspace of the full stimulus that modulates neuronal responses. The dimensionality of these features are further

reduced when projected into UMAP space. Encouragingly, the modulation power spectrum shows similar characteristics across the two feature extraction methods.

Reviewer Point 2.4— *I realize that the analysis itself is solid and interesting, but I find the conclusion at the end somewhat contrived :*

"The similar structure of auditory cortical receptive fields in the starling and in the mouse, specifically the fact that they have multiple excitatory and inhibitory subunits, and bearing in mind the ancient split between the synapsids and diapsids during amniote evolution, indicates that this property is likely a generic principle of natural sound representation".

First of all, "having multiple excitatory and inhibitory subunits", is not a profound similarity in any way, It is obvious that these higher cortical areas have such a structure as the authors point out at length in the Introduction and motivations. Second, that the similarly clustered features of the STRFs somehow reflects a generic principle of natural sound representation common to the mouse and birds is an unnecessary weak speculation.

Reply: We appreciate your feedback on the conclusion of our paper. We understand your concern that the conclusion may be contrived and we apologize for any confusion. We have revised the conclusion to remove the speculation about the similar structure of auditory cortical receptive fields being a generic principle of natural sound representation. Instead, we have focused on highlighting the important findings of our study, which demonstrate the similarity between the receptive fields in the starling and mouse and provide new insights into the processing of natural sounds in the auditory cortex. Thank you again for your feedback and we hope that the revised conclusion better reflects the results and significance of our research.

Reviewer Point 2.5— *So in short, I suggest that the authors expand on their analysis by reporting the results of 2 or 3 other (even much simpler purely linear) STRF methods and contrast then to the results of the features reported here where possible.*

Reply:

Thank you for your suggestion to expand our analysis. We have added new analyses comparing the prediction accuracy of spike-triggered covariance (STC) models with the MNE method (Fig. 7). This plot has been added to Figure 5 of the original manuscript. These results provide a clearer picture of the strengths and limitations of different STRF estimation methods and add depth to our findings. We have also included the features estimated from STC in the UMAP latent space projection, which was shown in Figure 6 of the original manuscript (Fig. 6 here). We believe that these additions will greatly enhance the reader's understanding of our research and will better address your concerns.

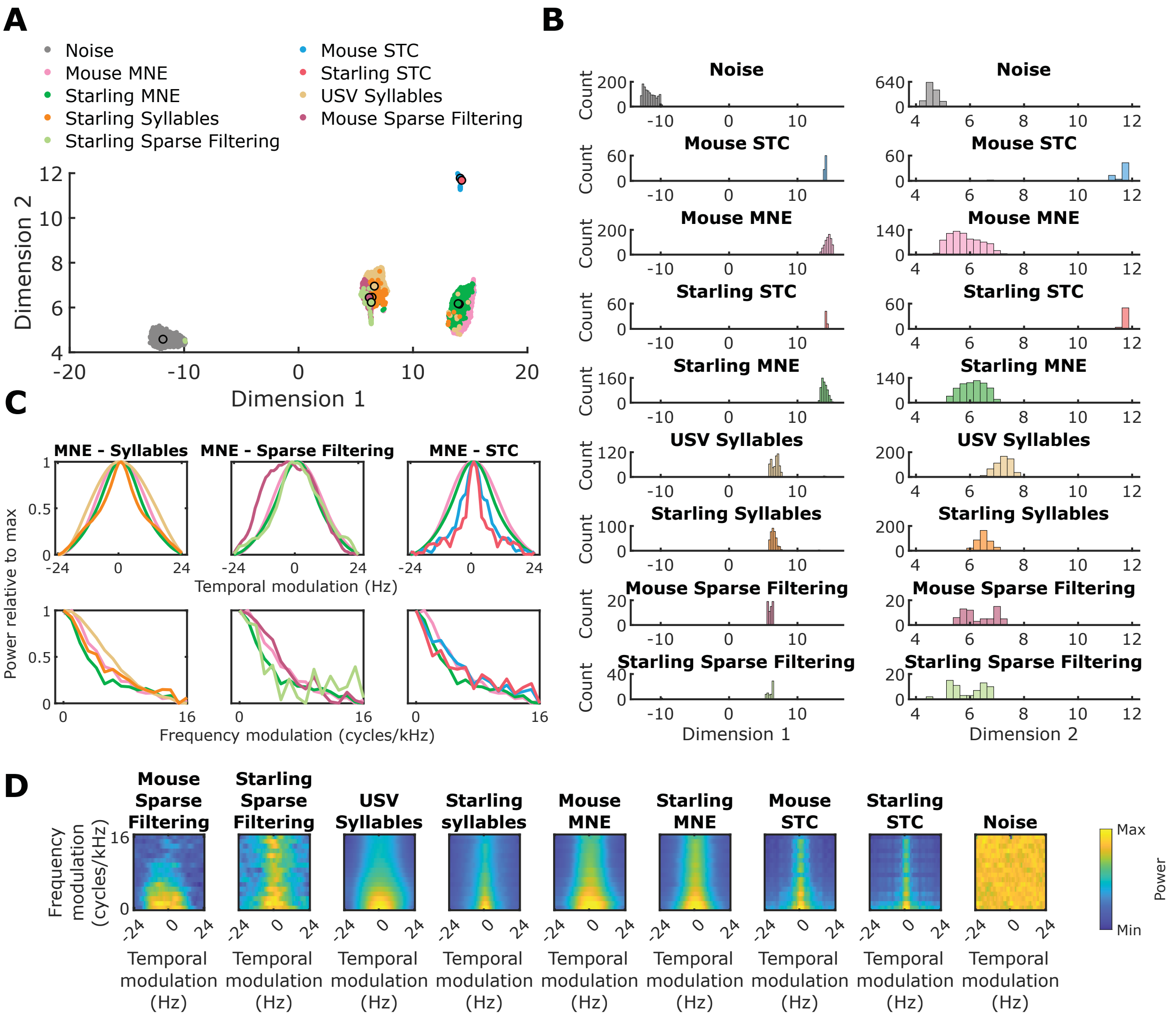

Figure 6: **(A)** UMAP projections onto two dimensions of: segmented syllables present in USVs and pitch-shifted starling songs, significant features estimated using the MNE models, features estimated using the sparse filtering method, and randomly generated noise. Larger points with outlines are the projection of the mean modulation power spectrum of the class as indicated by the label color. The leftmost cluster includes the USV and starling syllables segmented from the stimuli and the sparse filtering features estimated from the stimuli. The middle cluster is mainly comprised of the MNE features estimated from both stimulus types. The leftmost cluster clearly separates the noise. **(B)** Histograms of each of the different categories in dimension 1 (left) and dimension 2 (right). **(C)** Projections of modulation power spectra along the temporal axis (top) and spectral axis (bottom). Projection are all plotted as power relative to the maximum in order to facilitate comparison. The columns show the modulation spectra of MNE features in comparison with the syllables, sparse filtering features and STC features respectively. Colors are the same as in (A). **(D)** Mean modulation power spectra for each of the classes in (A).

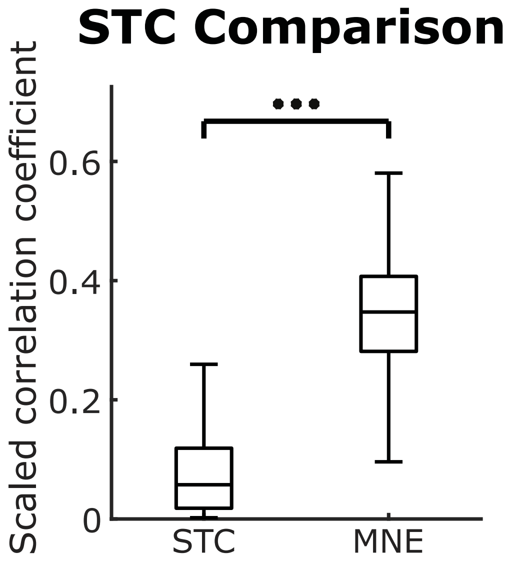

Figure 7: Comparison between the prediction accuracy of STC ($n = 128$, $\mu = 0.077$, $\sigma = 0.077$) and MNE ($n = 128$, $\mu = 0.34$, $\sigma = 0.11$) models. The scaled correlation coefficients for models fitted to both stimulus types were considered. $^{***}P < 0.001$ (paired t-test).

References

L. M. Aitkin and D. R. Moore. Inferior colliculus. II. Development of tuning characteristics and tonotopic organization in central nucleus of the neonatal cat. *Journal of Neurophysiology*, 38(5):1208–1216, Sept. 1975. ISSN 0022-3077. doi: 10.1152/jn.1975.38.5.1208.

L. M. Aitkin, W. R. Webster, J. L. Veale, and D. C. Crosby. Inferior colliculus. I. Comparison of response properties of neurons in central, pericentral, and external nuclei of adult cat. *Journal of Neurophysiology*, 38(5):1196–1207, Sept. 1975. ISSN 0022-3077. doi: 10.1152/jn.1975.38.5.1196.

C. A. Atencio and T. O. Sharpee. Multidimensional receptive field processing by cat primary auditory cortical neurons. *Neuroscience*, 359:130–141, Sept. 2017. ISSN 18737544. doi: 10.1016/j.neuroscience.2017.07.003. Publisher: Elsevier Ltd.

A. Calabrese, J. W. Schumacher, D. M. Schneider, L. Paninski, and S. M. N. Woolley. A Generalized Linear Model for Estimating Spectrotemporal Receptive Fields from Responses to Natural Sounds. *PLOS ONE*, 6(1):e16104, Jan. 2011. ISSN 1932-6203. doi: 10.1371/journal.pone.0016104. URL `https://journals.plos.org/plosone/article?id=10.1371/journal.pone.0016104`. Publisher: Public Library of Science.

M. B. Calford, W. R. Webster, and M. M. Semple. Measurement of frequency selectivity of single neurons in the central auditory pathway. *Hearing Research*, 11(3): 395–401, Sept. 1983. ISSN 0378-5955. doi: 10.1016/0378-5955(83)90070-9. URL `https://www.sciencedirect.com/science/article/pii/0378595583900709`.

S. V. David and J. L. Gallant. Predicting neuronal responses during natural vision. *Network: Computation in Neural Systems*, 16(2-3):239–260, Jan. 2005. ISSN 0954-898X. doi:

10.1080/09548980500464030. URL https://doi.org/10.1080/09548980500464030. Publisher: Taylor & Francis _eprint: https://doi.org/10.1080/09548980500464030.

R. C. deCharms, D. T. Blake, and M. M. Merzenich. Optimizing Sound Features for Cortical Neurons. *Science*, 280(5368):1439–1444, May 1998. doi: 10.1126/science.280.5368.1439. URL https://www.science.org/doi/full/10.1126/science.280.5368.1439. Publisher: American Association for the Advancement of Science.

D. A. Depireux, J. Z. Simon, D. J. Klein, and S. A. Shamma. Spectro-temporal response field characterization with dynamic ripples in ferret primary auditory cortex. *Journal of Neurophysiology*, 85(3): 1220–1234, Mar. 2001. ISSN 0022-3077. doi: 10.1152/jn.2001.85.3.1220.

A. J. Doupe. Song- and Order-Selective Neurons in the Songbird Anterior Forebrain and their Emergence during Vocal Development. *Journal of Neuroscience*, 17(3):1147–1167, Feb. 1997. ISSN 0270-6474, 1529-2401. doi: 10.1523/JNEUROSCI.17-03-01147.1997. URL https://www.jneurosci.org/content/17/3/1147. Publisher: Society for Neuroscience Section: Articles.

J. D. Fitzgerald, L. C. Sincich, and T. O. Sharpee. Minimal Models of Multidimensional Computations. *PLoS Computational Biology*, 7(3):e1001111, 2011. ISSN 1553-7358. doi: 10.1371/journal.pcbi.1001111. URL http://dx.plos.org/10.1371/journal.pcbi.1001111.

J. A. Grace, N. Amin, N. C. Singh, and F. E. Theunissen. Selectivity for conspecific song in the zebra finch auditory forebrain. *Journal of Neurophysiology*, 89(1):472–487, Jan. 2003. ISSN 0022-3077. doi: 10.1152/jn.00088.2002.

N. S. Harper, O. Schoppe, B. D. B. Willmore, Z. Cui, J. W. H. Schnupp, and A. J. King. Network Receptive Field Modeling Reveals Extensive Integration and Multi-feature Selectivity in Auditory Cortical Neurons. *PLOS Computational Biology*, 12(11): e1005113, Nov. 2016. ISSN 1553-7358. doi: 10.1371/journal.pcbi.1005113. URL https://journals.plos.org/ploscompbiol/article?id=10.1371/journal.pcbi.1005113. Publisher: Public Library of Science.

N. Kowalski, D. A. Depireux, and S. A. Shamma. Analysis of dynamic spectra in ferret primary auditory cortex. II. Prediction of unit responses to arbitrary dynamic spectra. *Journal of Neurophysiology*, 76 (5):3524–3534, Nov. 1996a. ISSN 0022-3077. doi: 10.1152/jn.1996.76.5.3524.

N. Kowalski, D. A. Depireux, and S. A. Shamma. Analysis of dynamic spectra in ferret primary auditory cortex. I. Characteristics of single-unit responses to moving ripple spectra. *Journal of Neurophysiology*, 76(5):3503–3523, Nov. 1996b. ISSN 0022-3077. doi: 10.1152/jn.1996.76.5.3503.

A. S. Kozlov and T. Q. Gentner. Central auditory neurons have composite receptive fields. *Proceedings of the National Academy of Sciences of the United States of America*, 113(5):1441–6, Feb. 2016. ISSN 1091-6490. doi: 10.1073/pnas.1506903113. URL http://www.pnas.org/cgi/content/long/113/5/1441.

J. Laudanski, J.-M. Edeline, and C. Huetz. Differences between Spectro-Temporal Receptive Fields Derived from Artificial and Natural Stimuli in the Auditory Cortex. *PLOS ONE*, 7(11):e50539, Nov. 2012. ISSN 1932-6203. doi: 10.1371/journal.pone.0050539. URL

https://journals.plos.org/plosone/article?id=10.1371/journal.pone.0050539. Publisher: Public Library of Science.

L.-y. Li, X. R. Xiong, L. A. Ibrahim, W. Yuan, H. W. Tao, and L. I. Zhang. Differential Receptive Field Properties of Parvalbumin and Somatostatin Inhibitory Neurons in Mouse Auditory Cortex. *Cerebral Cortex*, 25(7):1782–1791, July 2015. ISSN 1047-3211. doi: 10.1093/cercor/bht417. URL https://doi.org/10.1093/cercor/bht417.

J. F. Linden, R. C. Liu, M. Sahani, C. E. Schreiner, and M. M. Merzenich. Spectrotemporal structure of receptive fields in areas AI and AAF of mouse auditory cortex. *Journal of Neurophysiology*, 90(4): 2660–2675, Oct. 2003. ISSN 0022-3077. doi: 10.1152/jn.00751.2002.

C. K. Machens, M. S. Wehr, and A. M. Zador. Linearity of Cortical Receptive Fields Measured with Natural Sounds. *The Journal of Neuroscience*, 24(5):1089–1100, Feb. 2004. doi: 10.1523/jneurosci.4445-03.2004. MAG ID: 2131562784.

L. McInnes and J. Healy. Accelerated Hierarchical Density Based Clustering. *2017 IEEE International Conference on Data Mining Workshops (ICDMW)*, pages 33–42, Nov. 2017. doi: 10.1109/ICDMW.2017.12. URL http://ieeexplore.ieee.org/document/8215642/. Conference Name: 2017 IEEE International Conference on Data Mining Workshops (ICDMW) ISBN: 9781538638002 Place: New Orleans, LA Publisher: IEEE.

R. J. Rowekamp and T. O. Sharpee. Cross-orientation suppression in visual area V2. *Nature Communications*, 8:1–9, 2017. ISSN 20411723. doi: 10.1038/ncomms15739. URL http://dx.doi.org/10.1038/ncomms15739. Publisher: Nature Publishing Group.

T. Sainburg, M. Thielk, and T. Q. Gentner. Finding, visualizing, and quantifying latent structure across diverse animal vocal repertoires. *PLOS Computational Biology*, 16 (10):e1008228, Oct. 2020. ISSN 1553-7358. doi: 10.1371/journal.pcbi.1008228. URL https://journals.plos.org/ploscompbiol/article?id=10.1371/journal.pcbi.1008228. Publisher: Public Library of Science.

N. C. Singh and F. E. Theunissen. Modulation spectra of natural sounds and ethological theories of auditory processing. *J Acoust Soc Am*, 114(6 Pt 1):3394–3411, 2003. ISSN 00014966. doi: 10.1121/1.1624067. ISBN: 0001-4966 (Print)\r0001-4966 (Linking).

I. Stiebler, R. Neulist, I. Fichtel, and G. Ehret. The auditory cortex of the house mouse: left-right differences, tonotopic organization and quantitative analysis of frequency representation. *Journal of Comparative Physiology. A, Sensory, Neural, and Behavioral Physiology*, 181(6):559–571, Dec. 1997. doi: 10.1007/s003590050140.

F. E. Theunissen, K. Sen, and A. J. Doupe. Spectral-Temporal Receptive Fields of Nonlinear Auditory Neurons Obtained Using Natural Sounds. *Journal of Neuroscience*, 20(6):2315–2331, Mar. 2000. ISSN 0270-6474, 1529-2401. doi: 10.1523/JNEUROSCI.20-06-02315.2000. URL https://www.jneurosci.org/content/20/6/2315. Publisher: Society for Neuroscience Section: ARTICLE.

S. M. N. Woolley, P. R. Gill, and F. E. Theunissen. Stimulus-Dependent Auditory Tuning Results in Synchronous Population Coding of Vocalizations in the Songbird Midbrain. *Journal of Neuroscience*,

26(9):2499–2512, Mar. 2006. ISSN 0270-6474, 1529-2401. doi: 10.1523/JNEUROSCI.3731-05.2006. URL https://www.jneurosci.org/content/26/9/2499. Publisher: Society for Neuroscience Section: Articles.

Dear Dr Kozlov,

Re: JP-RP-2023-285003X "Composite receptive fields in the mouse auditory cortex" by Sihao Lu, Grace W. Ang, Mark Steadman, and Andrei S. Kozlov

Thank you for resubmitting your revised Research Article to The Journal of Physiology. It has been assessed by the original Reviewing Editor and Referees and has been well received. Some final revisions have been requested.

Please advise your co-authors of this decision as soon as possible.

The referee reports are copied at the end of this email.

Please address all the points raised and incorporate all requested revisions or explain in your Response to Referees why a change has not been made. We hope you will find the comments helpful and that you will be able to return your revised manuscript within 4 weeks. If you require longer than this, please contact journal staff: jp@physoc.org.

Your revised manuscript should be submitted online using the link in your Author Tasks: Link Not Available. This link is accessible via your account as Corresponding Author; it is not available to your co-authors. If this presents a problem, please contact journal staff (jp@physoc.org). Image files from the previous version are retained on the system. Please ensure you replace or remove any files that are being revised.

If you do not wish to submit a revised version of your manuscript, you must inform our journal staff (jp@physoc.org) or reply to this email to request withdrawal. Please note that a manuscript must be formally withdrawn from the peer review process at one journal before it may be submitted to another journal.

TRANSPARENT PEER REVIEW POLICY: To improve the transparency of its peer review process, The Journal of Physiology publishes online as supporting information the peer review history of all articles accepted for publication. Readers will have access to decision letters, including Editors' comments and referee reports, for each version of the manuscript, as well as any author responses to peer review comments. Referees can decide whether or not they wish to be named on the peer review history document.

ABSTRACT FIGURES: Authors are expected to use The Journal's premium BioRender account to create/redraw their Abstract Figures. Information on how to access this account is here: https://physoc.onlinelibrary.wiley.com/journal/14697793/biorender-access.

This will enable Authors to create and download high-resolution figures. If authors have used the free BioRender service, they can use the instructions provided in the link above to download a high-resolution version suitable for publication.

The link provided should only be used for the purposes of this submission. Authors will be charged for figures created on this account if they are not related to this manuscript submission.

LANGUAGE EDITING AND SUPPORT FOR PUBLICATION: If you would like help with English language editing, or other article preparation support, Wiley Editing Services offers expert help, including English Language Editing, as well as translation, manuscript formatting, and figure formatting at www.wileyauthors.com/eeo/preparation. You can also find resources for Preparing Your Article for general guidance about writing and preparing your manuscript at www.wileyauthors.com/eeo/prepresources.

REVISION CHECKLIST:

Check that your Methods section conforms to journal policy: https://jp.msubmit.net/cgi-bin/main.plex?form_type=display_requirements#methods.

Check that data presented conforms to the statistics policy: https://jp.msubmit.net/cgi-bin/main.plex?form_type=display_requirements#statistics.

Upload a full Response to Referees file. To create your 'Response to Referees' copy all the reports, including any comments from the Senior and Reviewing Editors, into a Microsoft Word, or similar, file and respond to each point, using font or background colour to distinguish comments and responses and upload as the required file type.

Please upload two versions of your manuscript text: one with all relevant changes highlighted and one clean version with no changes tracked. The manuscript file should include all tables and figure legends, but each figure/graph should be uploaded as separate, high-resolution files.

You may also upload:

- 'Potential Cover Art' for consideration as the issue's cover image

- Appropriate Supporting Information (Video, audio or data set: see https://jp.msubmit.net/cgi-bin/main.plex?form_type=display_requirements#supp).

We look forward to receiving your revised submission.

If you have any queries, please reply to this email and we will be pleased to advise.

Yours sincerely,

Richard Carson
Senior Editor
The Journal of Physiology

REQUIRED ITEMS FOR REVISION

-Include a Key Points list in the article itself, before the Abstract.

-You must start the Methods section with a paragraph headed Ethical Approval. A detailed explanation of journal policy and regulations on animal experimentation is given in Principles and standards for reporting animal experiments in The Journal of Physiology and Experimental Physiology by David Grundy J Physiol, 593: 2547-2549. doi:10.1113/JP270818.). A checklist outlining these requirements and detailing the information that must be provided in the paper can be found at: https://physoc.onlinelibrary.wiley.com/hub/animal-experiments. Authors should confirm in their Methods section that their experiments were carried out according to the guidelines laid down by their institution's animal welfare committee, and conform to the principles and regulations as described in the Editorial by Grundy (2015). The Methods section must contain details of the anaesthetic regime: anaesthetic used, dose and route of administration and method of killing the experimental animals.

-Your manuscript must include a complete Additional Information section

-The Journal of Physiology funds authors of provisionally accepted papers to use the premium BioRender site to create high resolution schematic figures. Follow this link and enter your details and the manuscript number to create and download figures. Upload these as the figure files for your revised submission. If you choose not to take up this offer we require figures to be of similar quality and resolution. If you are opting out of this service to authors, state this in the Comments section on the Detailed Information page of the submission form. The link provided should only be used for the purposes of this submission. Authors will be charged for figures created on this premium BioRender account if they are not related to this manuscript submission.

-Please ensure that the Article File you upload is a Word file.

-Papers must comply with the Statistics Policy https://jp.msubmit.net/cgi-bin/main.plex?form_type=display_requirements#statistics

In summary:

-If n {less than or equal to} 30, all data points must be plotted in the figure in a way that reveals their range and distribution. A bar graph with data points overlaid, a box and whisker plot or a violin plot (preferably with data points included) are acceptable formats.

-If n > 30, then the entire raw dataset must be made available either as supporting information, or hosted on a not-for-profit repository e.g. FigShare, with access details provided in the manuscript.

-'n' clearly defined (e.g. x cells from y slices in z animals) in the Methods. Authors should be mindful of pseudoreplication.

-All relevant 'n' values must be clearly stated in the main text, figures and tables, and the Statistical Summary Document

(required upon revision)

-The most appropriate summary statistic (e.g. mean or median and standard deviation) must be used. Standard Error of the Mean (SEM) alone is not permitted.

-Exact p values must be stated. Authors must not use 'greater than' or 'less than'. Exact p values must be stated to three significant figures even when 'no statistical significance' is claimed.

-Statistics Summary Document completed appropriately upon revision

-A Data Availability Statement is required for all papers reporting original data. This must be in the Additional Information section of the manuscript itself. It must have the paragraph heading "Data Availability Statement". All data supporting the results in the paper must be either: in the paper itself; uploaded as Supporting Information for Online Publication; or archived in an appropriate public repository. The statement needs to describe the availability or the absence of shared data. Authors must include in their Statement: a link to the repository they have used, or a statement that it is available as Supporting Information; reference the data in the appropriate sections(s) of their manuscript; and cite the data they have shared in the References section. Whenever possible the scripts and other artefacts used to generate the analyses presented in the paper should also be publicly archived. If sharing data compromises ethical standards or legal requirements then authors are not expected to share it, but must note this in their Statement. For more information, see our Statistics Policy.

-Please include an Abstract Figure file, as well as the figure legend text within the main article file. The Abstract Figure is a piece of artwork designed to give readers an immediate understanding of the research and should summarise the main conclusions. If possible, the image should be easily 'readable' from left to right or top to bottom. It should show the physiological relevance of the manuscript so readers can assess the importance and content of its findings. Abstract Figures should not merely recapitulate other figures in the manuscript. Please try to keep the diagram as simple as possible and without superfluous information that may distract from the main conclusion(s). Abstract Figures must be provided by authors no later than the revised manuscript stage and should be uploaded as a separate file during online submission labelled as File Type 'Abstract Figure'. Please ensure that you include the figure legend in the main article file. All Abstract Figures should be created using BioRender. Authors should use The Journal's premium BioRender account to export high-resolution images. Details on how to use and access the premium account are included as part of this email.

-Please include a full title page as part of your article (Word) file (containing title, authors, affiliations, corresponding author name and contact details, keywords, and running title).

EDITOR COMMENTS

Reviewing Editor:

Ethics Concerns:
The manuscript should include details on: the origin of the animals used in the experiments, their access to food and water, their eventual fate (presumably euthanasia at the end of the recording?), how the level of anaesthesia was measured and managed throughout the recordings.

Comments for Authors to ensure the paper complies with the Statistics Policy:
Multiple panels in several figures are shown as box plots. The journal standards require that the individual points are shown or if n>30 (which is the case here) and the individual data points are not shown, they are provided as supporting information files.

Comments to the Author:
Thank you for resubmitting the manuscript. The revised version was evaluated by the two original reviewers who were positive about the revision. One of the reviewers raised a few points that still need to be addressed. In addition, the manuscript needs to include ethics and statistics details as required by the journal's policies.

Senior Editor:

Comments for Authors to ensure the paper complies with the Statistics Policy:

Please refer to the Statistics Policy of the Journal.

In this regard, the Reviewing Editor has highlighted one specific issue concerning the use of box plots.

Whenever it is feasible to do so, individual data points should be shown.

In addition, asterisks should not be be used on figures to indicate contrasts that are deemed to be "statistically significant".

REFEREE COMMENTS

Referee #1:

I thank the authors for implementing comments, providing explanations, and revising the manuscript.

It is much improved and below I detail a few points that, I believe, are important to address:

1. How good is the model fit to the data?

The measure of goodness-of-fit used by the authors is a relative one. Meaning, that it tells us how much variance is explained from all the variance that could be explained (if trials are identical).

This is confusing. Calabrese et al. 2011 and David&Gallant 2005 seem to use different normalizations. Is there a source that used the same normalization (scaling)?

Put differently,

The discussion states "However, given the relatively low predictive power of the MNE models with these data, it would be important to investigate this question using better models of complex receptive fields". The normalized correlation measure does not allow the reader to know what does "low predictive power" mean? How low?

I really suggest to provide the actual, not-normalized, performance of the model up front. Then, the normalized measure can be justified and used.

2. Similarity of NME features between USVs and starling syllables.

It would be very useful to show features that look similar as examples.

The UMAP technique tells us that the NME features cluster together. This tells us that the features are close but does not give intuition about the feature shapes.

Also, was the noise input also used when fitting the UMAP?

If so, it would be good to see the fit without the noise since it can really bias the results.

3. The linear response.

If I understand correctly, only quadratic features were displayed. What was the predictive power of the linear part of the MNE? How does it compare with the quadratic? Do these (linear) features resemble between the USVs and starling song?

Referee #2:

The paper has been adequately revised and the authors have answered all my questions and seems to be also the case of the other reviewer's questions.

END OF COMMENTS

Response letter

We are grateful to the editors and reviewers for their time to reassess our modified manuscript. We are glad to learn that it has been well received. Below we will provide a comprehensive account of the additions to the manuscript addressing the final requested revisions point-by-point.

Editor 1

Editor Point 1.1— *Ethics Concerns: The manuscript should include details on: the origin of the animals used in the experiments, their access to food and water, their eventual fate (presumably euthanasia at the end of the recording?), how the level of anaesthesia was measured and managed throughout the recordings.*

Reply: We have included a new *Ethics statement* section to the *Materials and Methods* which we copy here:

"All procedures were carried out under the terms and conditions of licences issued by the UK Home Office under the Animals (Scientific Procedures) Act 1986. Adult female C57BL/6 mice (Charles River, Margate, UK) were used for electrophysiological recordings (N=25, aged 5 to 27 weeks). All mice were housed in groups of five and allowed food and water *ad libitum*. Animals were humanely killed in accordance with Schedule 1 to the Animals (Scientific Procedures) Act 1986. "

In addition, we clarified the measurement of the level of anaesthesia in the *Surgical preparation* section: "Throughout the entirety of the surgical procedure and the electrical recording, the depth of anaesthesia was continuously monitored through the breathing rate and periodic assessment of the toe pinch reflex."

Editor Point 1.2— *Comments for Authors to ensure the paper complies with the Statistics Policy: Multiple panels in several figures are shown as box plots. The journal standards require that the individual points are shown or if $n > 30$ (which is the case here) and the individual data points are not shown, they are provided as supporting information files.*

Reply: We have now included all the data used for the box- and scatter plots under the 'Supporting Information for online publication' section of the submission portal.

Editor Point 1.3— *Comments to the Author: Thank you for resubmitting the manuscript. The revised version was evaluated by the two original reviewers who were positive about the revision. One of the reviewers raised a few points that still need to be addressed. In addition, the manuscript needs to include ethics and statistics details as required by the journal's policies.*

Reply: We are grateful that the revised manuscript was received positively. Below we will detail the additional changes made at the request of the reviewers in a point-by-point fashion. As mentioned in point 1.1 and 1.2, we have included a new *Ethics statement* section and have revised the presentation of our statistics to comply with the journal's Statistics Policy.

Editor 2

Editor Point 2.1— *Comments for Authors to ensure the paper complies with the Statistics Policy: Please refer to the Statistics Policy of the Journal. In this regard, the Reviewing Editor has highlighted one specific issue concerning the use of box plots.*

Whenever it is feasible to do so, individual data points should be shown. In addition, asterisks should not be be used on figures to indicate contrasts that are deemed to be "statistically significant".

Reply: We have revised the figures to include the exact P values directly on the plots instead of the asterisks and we have included all the data used for the box- and scatter plots under the 'Supporting Information for online publication' section of the submission portal.

Reviewer 1

Reviewer Point 1.1— *I thank the authors for implementing comments, providing explanations, and revising the manuscript. It is much improved and below I detail a few points that, I believe, are important to address:*

Reply: We are glad to hear that the reviewer thinks the revised manuscript is much improved. We will address each of the highlighted points below.

Reviewer Point 1.2— *1. How good is the model fit to the data? The measure of goodness-of-fit used by the authors is a relative one. Meaning, that it tells us how much variance is explained from all the variance that could be explained (if trials are identical). This is confusing. Calabrese et al. 2011 and David & Gallant 2005 seem to use different normalizations. Is there a source that used the same normalization (scaling)?*

Put differently, The discussion states "However, given the relatively low predictive power of the MNE models with these data, it would be important to investigate this question using better models of complex receptive field". The normalized correlation measure does not allow the reader to know what does "low predictive power" mean? How low? I really suggest to provide the actual, not-normalized, performance of the model up front. Then, the normalized measure can be justified and used.

Reply: We do provide the non-normalized measure (Figure 5 B and E). Namely, we plot the non-normalized correlation coefficients against the normalized ones (called correlation coefficients upper bound). The source of this approach is described, along with the approach itself, at the end of the *Receptive field characterisation* section in *Materials and Methods*. We have now added emphasis to that text: "Both raw and scaled correlation coefficients were plotted for each unit".

In addition, it is our understanding that the normalization mentioned by both David and Gallant [2005] and Calabrese et al. [2011] that the reviewer is referring to is the normalized reverse correlation (NRC). This is a method developed to address the limitation of classical spectro-temporal receptive field (STRF) methods which assume that the stimuli presented are circularly symmetric (i.e., there are no stimulus correlations, which is not the case for natural stimuli). When this assumption is not met, the STRFs obtained using classical methods can be biased. NRC minimizes this bias by using

an approximation to the stimulus covariance matrix to normalize the estimated STRF. Calabrese et al. [2011] point to other methods that address this issue, including Maximally Informative Dimensions (MID, [Sharpee et al., 2004]) which is based on information theory, similar to the method used in our manuscript. Crucially, the normalization employed by David and Gallant [2005] and Calabrese et al. [2011] does not address inherent variability of the neural activity on a trial-to-trial basis. David and Gallant [2005] highlight this in their work and point the reader to the method employed in our work ([Hsu et al., 2004]): "Although accurate measurement of experimental noise is crucial for assessing and comparing alternative models objectively, this issue has received little attention thus far (but see Sahani & Linden 2003; Hsu et al. 2004)." Lastly, we have included several other works using this method on electrophysiological data from auditory cortex in the reference section of this document [Rahman et al., 2019, Espejo et al., 2019, Elie and Theunissen, 2019, Shih et al., 2020].

Reviewer Point 1.3— *2. Similarity of NME features between USVs and starling syllables.*

It would be very useful to show features that look similar as examples. The UMAP technique tells us that the NME features cluster together. This tells us that the features are close but does not give intuition about the feature shapes. Also, was the noise input also used when fitting the UMAP? If so, it would be good to see the fit without the noise since it can really bias the results.

Reply: We thank the reviewer for their input and understand that it would be helpful to provide examples of similar features. In Figure 1 we show a comparison between the features that are the most similar and least similar for MNE models derived from the two types of stimuli (USV and pitch-shifted starling song) (Figure 1A and B). In addition, we also show the most and least similar features between the two classes (Figure 1C). The similarity in all instances is defined as the Euclidean distance in the UMAP latent space.

In Figure 2, we plot the results of a UMAP fit without the noise samples. As in the previous result, the MNE and STC features both form their own clusters whilst the syllables and the sparse filtering features cluster together.

Reviewer Point 1.4— *3. The linear response.*

If I understand correctly, only quadratic features were displayed. What was the predictive power of the linear part of the MNE? How does it compare with the quadratic? Do these (linear) features resemble between the USVs and starling song?

Reply:

The reviewer is correct in assuming that only the quadratic features are displayed. In Figure 3 in this document, we show the predictive power of using the full model as compared to using only the linear component of the model for both types of stimuli.

Unlike the quadratic features, the linear features show little appreciable structure for both the USV and pitch-shifted starling song stimuli (Figure 4).

Reviewer 2

Reviewer Point 2.1— *The paper has been adequately revised and the authors have answered all my questions and seems to be also the case of the other reviewer's questions.*

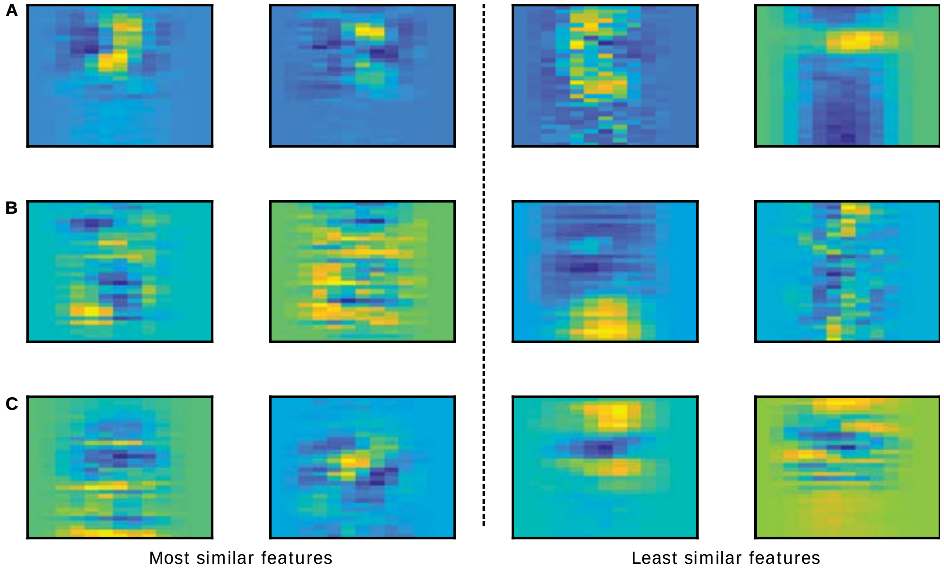

Figure 1: *Comparison of most and least similar features between classes.* **(A)** Two most similar features from MNE models trained on USVs (left) compared against the two least similar features in the same class (right). **(B)** Two most similar features from MNE models trained on pitch-shifted starling song (left) compared against the two least similar features in the same class (right). **(C)** Two most similar features between MNE models trained on USVs and pitch-shifted starling song (left) compared against the two least similar features between the two classes (right). Similarity as measured by Euclidean distance in the UMAP latent space.

Reply: We would like to reiterate our appreciation of the reviewer's valuable suggestions, as they have significantly enhanced the quality of our manuscript. We are glad that our revisions adequately addressed their concerns.

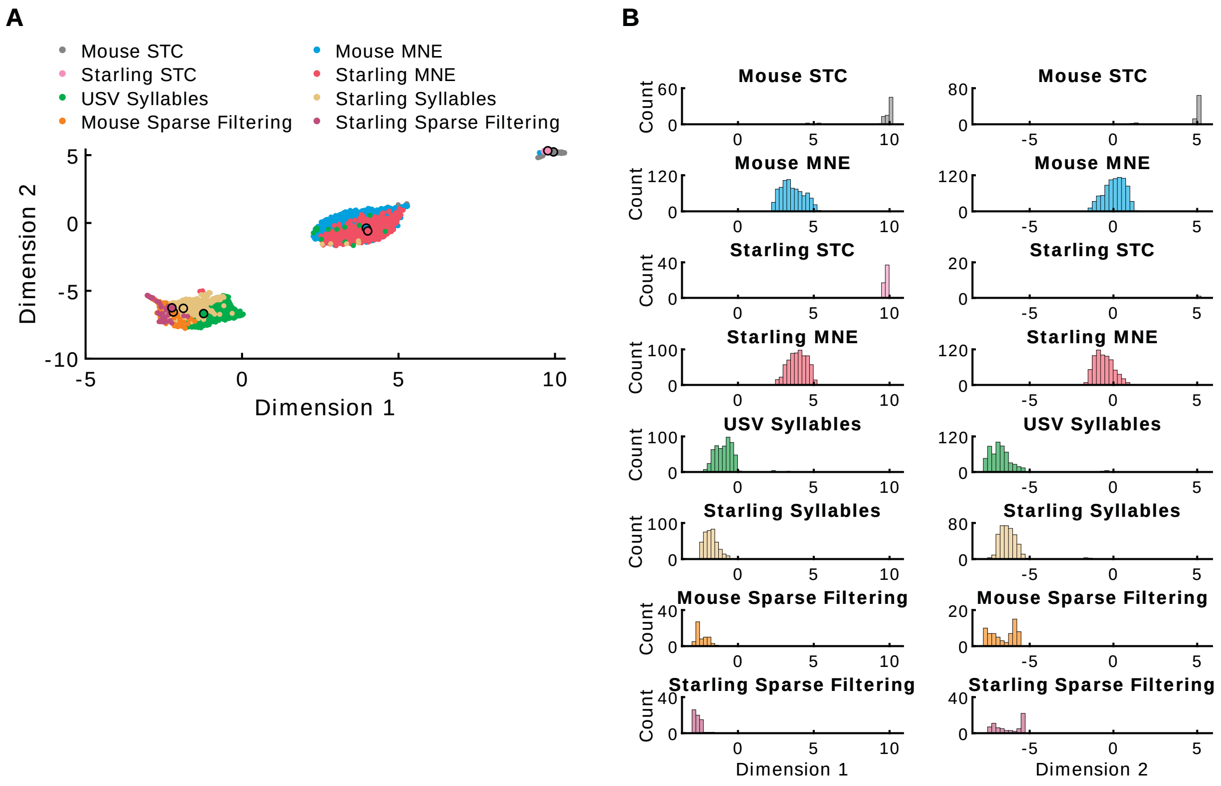

Figure 2: **(A)** UMAP projections onto two dimensions of: segmented syllables present in USVs and pitch-shifted starling songs, significant features estimated using the MNE models, features estimated using the sparse filtering method, and randomly generated noise. Larger points with outlines are the projection of the mean modulation power spectrum of the class as indicated by the label color. The leftmost cluster includes the USV and starling syllables segmented from the stimuli and the sparse filtering features estimated from the stimuli. The middle cluster is mainly comprised of the MNE features estimated from both stimulus types. **(B)** Histograms of each of the different categories in dimension 1 (left) and dimension 2 (right).

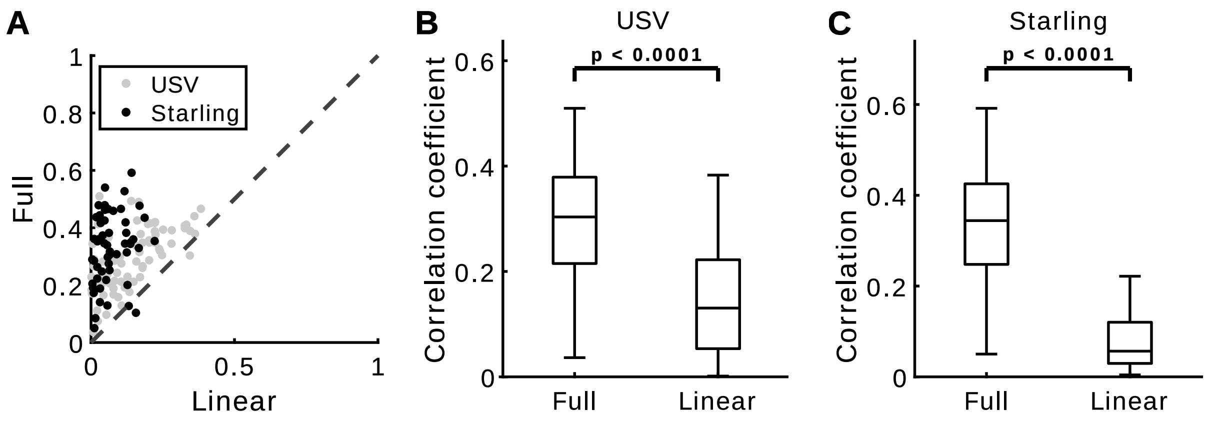

Figure 3: *Comparison between linear and quadratic model performance.* (**A**) Quadratic prediction correlation coefficients plotted as a function of the prediction performance when only using the linear component of the model. Two sets of points are shown; USVs (grey) and pitch-shifted starling song (black). Dashed line indicates a gradient of 1. (**B**) Comparison between the prediction accuracy of the full quadratic MNE model ($\mu = 0.299, \sigma = 0.104$) versus only the linear component ($\mu = 0.144, \sigma = 0.105$) for units responding to USV ($n = 74$). $p < 0.0001$ (paired t-test). (**C**) Comparison between the prediction accuracy of the full quadratic MNE model ($\mu = 0.328, \sigma = 0.125$) versus only the linear component ($\mu = 0.071, \sigma = 0.055$) for units responding to starling song ($n = 54$). $p < 0.0001$ (paired t-test). The correlation coefficients shown here are not scaled.

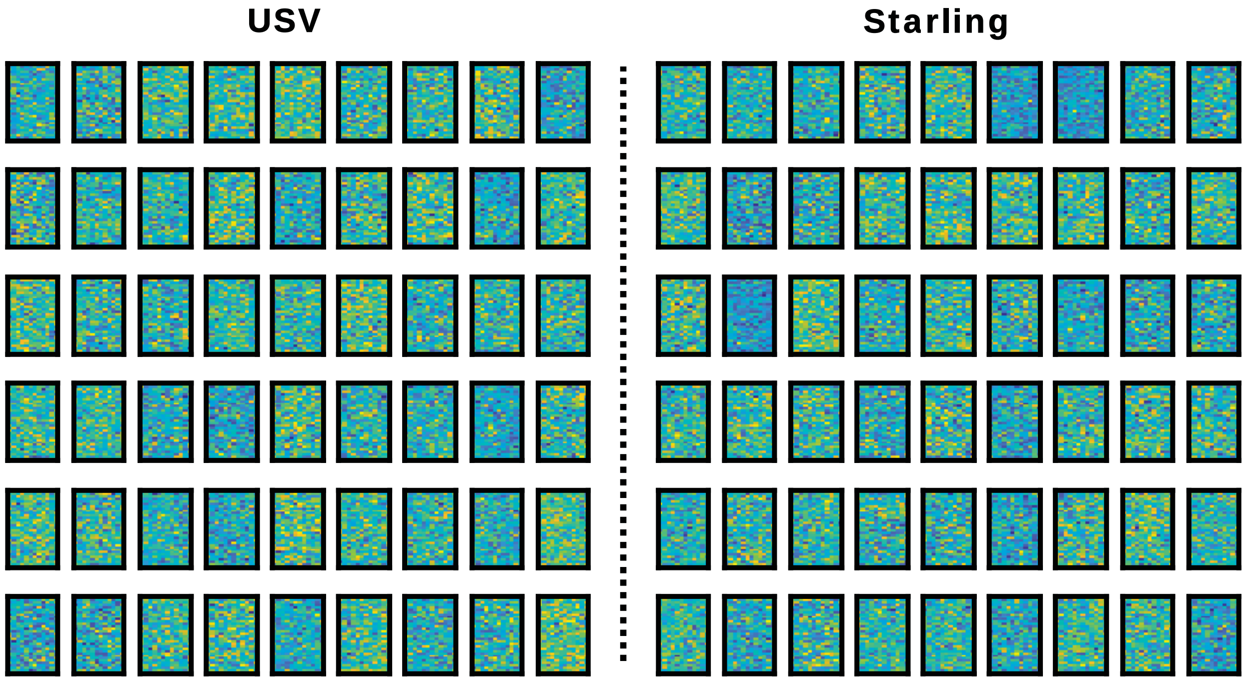

Figure 4: *Comparison between the linear features.* The linear features of MNE models fitted with USVs (left) and the corresponding linear features of the models fitted with pitch-shifted starling song (right).

References

A. Calabrese, J. W. Schumacher, D. M. Schneider, L. Paninski, and S. M. N. Woolley. A Generalized Linear Model for Estimating Spectrotemporal Receptive Fields from Responses to Natural Sounds. *PLOS ONE*, 6(1):e16104, Jan. 2011. ISSN 1932-6203. doi: 10.1371/journal.pone.0016104. URL `https://journals.plos.org/plosone/article?id=10.1371/journal.pone.0016104`. Publisher: Public Library of Science.

S. V. David and J. L. Gallant. Predicting neuronal responses during natural vision. *Network: Computation in Neural Systems*, 16(2-3):239–260, Jan. 2005. ISSN 0954-898X. doi: 10.1080/09548980500464030. URL `https://doi.org/10.1080/09548980500464030`. Publisher: Taylor & Francis _eprint: https://doi.org/10.1080/09548980500464030.

J. E. Elie and F. E. Theunissen. Invariant neural responses for sensory categories revealed by the time-varying information for communication calls. *PLOS Computational Biology*, 15(9):e1006698, Sept. 2019. ISSN 1553-7358. doi: 10.1371/journal.pcbi.1006698. URL `https://journals.plos.org/ploscompbiol/article?id=10.1371/journal.pcbi.1006698`. Publisher: Public Library of Science.

M. L. Espejo, Z. P. Schwartz, and S. V. David. Spectral tuning of adaptation supports coding of sensory context in auditory cortex. *PLOS Computational Biology*, 15 (10):e1007430, Oct. 2019. ISSN 1553-7358. doi: 10.1371/journal.pcbi.1007430. URL `https://journals.plos.org/ploscompbiol/article?id=10.1371/journal.pcbi.1007430`. Publisher: Public Library of Science.

A. Hsu, A. Borst, and F. E. Theunissen. Quantifying variability in neural responses and its application for the validation of model predictions. *Network: Computation in Neural Systems*, 15(2): 91–109, Jan. 2004. ISSN 0954-898X.

M. Rahman, B. D. B. Willmore, A. J. King, and N. S. Harper. A dynamic network model of temporal receptive fields in primary auditory cortex. *PLOS Computational Biology*, 15(5):e1006618, May 2019. ISSN 1553-7358. doi: 10.1371/journal.pcbi.1006618. URL `https://journals.plos.org/ploscompbiol/article?id=10.1371/journal.pcbi.1006618`. Publisher: Public Library of Science.

T. O. Sharpee, N. C. Rust, and W. Bialek. Analyzing neural responses to natural signals: maximally informative dimensions. *Neural computation*, 16(2), Feb. 2004. ISSN 0899-7667. doi: 10.1162/089976604322742010. URL `https://pubmed.ncbi.nlm.nih.gov/15006095/`. Publisher: Neural Comput.

J. Y. Shih, K. Yuan, C. A. Atencio, and C. E. Schreiner. Distinct Manifestations of Cooperative, Multidimensional Stimulus Representations in Different Auditory Forebrain Stations. *Cerebral Cortex*, 30(5):3130–3147, May 2020. ISSN 1047-3211. doi: 10.1093/cercor/bhz299. URL `https://doi.org/10.1093/cercor/bhz299`.

2nd Revision - Editorial Decision 12-Jul-2023

Dear Dr Kozlov,

Re: JP-RP-2023-285003XR1 "Composite receptive fields in the mouse auditory cortex" by Sihao Lu, Grace W. Ang, Mark Steadman, and Andrei S. Kozlov

We are pleased to tell you that your paper has been accepted for publication in The Journal of Physiology.

TRANSPARENT PEER REVIEW POLICY: To improve the transparency of its peer review process, The Journal of Physiology publishes online as supporting information the peer review history of all articles accepted for publication. Readers will have access to decision letters, including Editors' comments and referee reports, for each version of the manuscript, as well as any author responses to peer review comments. Referees can decide whether or not they wish to be named on the peer review history document.

The last Word (or similar) version of the manuscript provided will be used by the Production Editor to prepare your proof. When this is ready you will receive an email containing a link to Wiley's Online Proofing System. The proof should be thoroughly checked and corrected as promptly as possible.

Authors should note that it is too late at this point to offer corrections prior to proofing. The accepted version will be published online, ahead of the copy edited and typeset version being made available. Major corrections at proof stage, such as changes to figures, will be referred to the Editors for approval before they can be incorporated. Only minor changes, such as to style and consistency, should be made at proof stage. Changes that need to be made after proof stage will usually require a formal correction notice.

All queries at proof stage should be sent to: TJP@wiley.com.

Are you on Twitter? Once your paper is online, why not share your achievement with your followers? Please tag The Journal (@jphysiol) in any tweets and we will share your accepted paper with our 30,000 followers!

Yours sincerely,

Richard Carson
Senior Editor
The Journal of Physiology

P.S. - You can help your research get the attention it deserves! Check out Wiley's free Promotion Guide for best-practice recommendations for promoting your work at www.wileyauthors.com/eeo/guide. You can learn more about Wiley Editing Services which offers professional video, design, and writing services to create shareable video abstracts, infographics, conference posters, lay summaries, and research news stories for your research at www.wileyauthors.com/eeo/promotion.

IMPORTANT NOTICE ABOUT OPEN ACCESS: To assist authors whose funding agencies mandate public access to published research findings sooner than 12 months after publication, The Journal of Physiology allows authors to pay an Open Access (OA) fee to have their papers made freely available immediately on publication.

The Corresponding Author will receive an email from Wiley with details on how to register or log-in to Wiley Authors Services where you will be able to place an order.

You can check if your funder or institution has a Wiley Open Access Account here: https://authorservices.wiley.com/author-resources/Journal-Authors/licensing-and-open-access/open-access/author-compliance-tool.html.

REVIEWING EDITOR COMMENTS

Thank you for addressing the points raised by the editors and the reviewers.

2nd Confidential Review 05-Jul-2023
